# Running the Risk: Road‐Crossing Behavior in Wild Chimpanzees (*Pan troglodytes*) in an Anthropogenic Habitat in Uganda

**DOI:** 10.1002/ajp.70000

**Published:** 2025-02-08

**Authors:** Marie Tellier, François Druelle, Marie Cibot, Johnmary Baruzaliire, Tom Sabiiti, Matthew R. McLennan

**Affiliations:** ^1^ Oniris ‐ Ecole Nationale Vétérinaire de Nantes, Agroalimentaire et de l'Alimentation Nantes France; ^2^ Bulindi Chimpanzee and Community Project Hoima Uganda; ^3^ UMR 7268 ADES, Aix‐Marseille Université, CNRS, EFS Marseille France; ^4^ Functional Morphology Laboratory University of Antwerp Antwerp Belgium; ^5^ Solâme Lorient France; ^6^ Faculty of Humanities and Social Sciences Oxford Brookes University Oxford UK

**Keywords:** anthropogenic habitat, behavioral flexibility, *Pan troglodytes*, risk perception, road crossing, roads

## Abstract

Recent research highlights the behavioral flexibility of wild chimpanzees in response to human‐induced changes in their environment, including agricultural and infrastructural development. The expansion of road networks threatens chimpanzee populations across Africa. Studying their road‐crossing behavior, especially outside protected areas where road impacts are greatest, helps identify factors influencing their choices and flexibility. This study seeks to gain a deeper understanding of how chimpanzees navigate busy roads and assess the danger posed by roads. Such insights are needed to develop effective conservation strategies in regions facing escalating human impact, including recommendations for the design and management of traffic on existing and future roads. Using a dataset of 129 video‐recorded road crossings spanning 38 months, we analyzed the behavioral adjustments of chimpanzees in Bulindi, Uganda, when crossing a recently paved, busy main road within their home range. Using generalized linear mixed models, we investigated chimpanzee risk perception, protective and cooperative behaviors, vigilance, and progression order during road crossings. We identified variations in their behavior according to age‐sex of individuals, group composition, and level of risk. We found that Bulindi chimpanzees exhibit behavioral strategies to reduce risks of collision or close encounters with humans on the road, as previously described. However, they were less vigilant than expected. We suggest that the chimpanzees have developed tolerance of the risks presented by the road, owing to their long history of crossing it before it was tarmacked and widened, and their familiarity with local people and motor traffic. Our results provide further evidence of the flexibility of wild chimpanzees. However, road crossings remain highly risky for large mammals like great apes, necessitating measures to mitigate the impact of road development on this and other endangered species (e.g. speed bumps, police enforcement, public awareness raising).

## Summary

- The expansion of road networks presents a major threat to wild chimpanzees.
- We studied chimpanzee behavior during 129 recorded road crossings at Bulindi, Uganda. We found behavioral patterns varied by level of risk, group composition and age‐sex of individuals.
- Bulindi chimpanzees show behavioral strategies to reduce risks during road crossings. Yet the chimpanzees were less vigilant than expected, suggesting behavioral tolerance toward the road.

## Graphical abstract

Video screenshot showing a mature male chimpanzee (*Pan troglodytes*) crossing the Hoima‐Masindi road in western Uganda, narrowly avoiding a car, with nearby onlookers (electricity workers ) and motorcyclists).

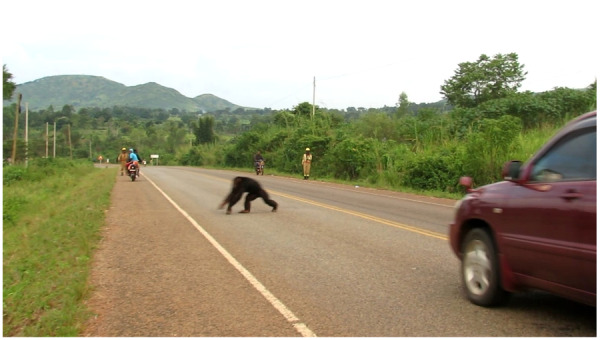

**Abbreviations**
AIC: Akaike Information Criteria
ANOVA: Analysis of Variance
BCCP: Bulindi Chimpanzee and Community Project
BORIS: Behavioral Observation Research Interactive Software
GLMM: Generalized Linear Mixed Model

## 1 Introduction

Human impact on the environment and the stability of ecosystems continues to expand through activities including logging, mining, farming and infrastructure development. In this context, most wild chimpanzee (*Pan troglodytes*) habitats in Africa have been human‐transformed in past decades, leading to a drastic decline in their populations (Plumptre et al. 2010; Humle et al. 2016; Kühl et al. 2017). A marker of human encroachment on wildlife habitats is the expansion of road networks, and a 35%–60% increase over the 2010 global road network length is expected by 2050 (Dulac 2013). Roads adversely affect wildlife survival across multiple levels (Jaeger et al. 2005; Bennett 2017). Chimpanzees are no exception. For example, only 4.3% of the western chimpanzees' geographical range is currently unimpacted by the presence of roads (Andrasi et al. 2021), and the same can be imagined for other chimpanzee subspecies considering the rapid development occurring over much of tropical Africa (Arcus Foundation 2018). Therefore, while road construction supports economic development across the continent (Laporte et al. 2007; Fayissa, Nsiah, and Tadasse 2008; Weng et al. 2013), it is also a threat to endangered mammals like chimpanzees because roads divide and fragment their habitats (e.g. Garriga et al. 2019; McLennan et al. 2021), facilitate poachers' access to their territories (Laurance et al. 2006; Vanthomme et al. 2013; Perumal et al. 2021), increase health risks due to plastic and atmospheric pollution (Krief et al. 2020) and zoonosis transmission (Namusisi et al. 2021), and expose them to the risk of collision with motorized traffic (McLennan and Asiimwe 2016; Krief et al. 2020; McLennan et al. 2021). In that respect, it is necessary to understand how wild chimpanzees adapt behaviorally to road and traffic infrastructures in their home range to better characterize the impact roads have on this great ape species and to assess their long‐term ability to persist in environments with roads. Such insights are urgently needed to implement or adapt conservation measures for this endangered species, as applied to traffic control and road design and construction (Laurance et al. 2014; Perumal et al. 2021).

Recent research has highlighted the behavioral flexibility of chimpanzees, including their responses to anthropogenic habitat changes (Hockings et al. 2015; McLennan, Spagnoletti, and Hockings 2017). For example, chimpanzees readily incorporate agricultural crops into their diets (e.g. McLennan 2013; Hockings, Anderson, and Matsuzawa 2012; Hockings and McLennan 2012; Couturier et al. 2022) and use exotic and planted species for nest construction (McCarthy, Lester, and Stanford 2017; McLennan et al. 2021). In “risky” habitats or situations, chimpanzees may modify their grouping patterns by moving in smaller subgroups (Satsias et al. 2022), adjust their activity budget by reducing resting time (Hockings, Anderson, and Matsuzawa 2012), nest more cohesively (van Dijk, Cibot, and McLennan 2021), modify their vocal behavior by vocalizing less often in areas with high human presence (Wilson, Hauser, and Wrangham 2007; Hicks, Roessingh, and Menken 2013), or shift their crop‐feeding activities to nighttime (Krief et al. 2014; Lacroux et al. 2022) to reduce the risk of encountering humans. These behavioral adjustments also include responses to novel situations that carry potential risks to health and survival such as increased contact with people (McLennan and Hill 2010) and domestic animals (Fryns et al. 2021), or the presence of roads to cross (Hockings, Anderson, and Matsuzawa 2006).

Paved and unpaved roads run through the ranges of various studied chimpanzee populations, in both protected (e.g. Cantanhez National Park in Guinea‐Bissau: Hockings and Sousa 2013; Bersacola, Hill, and Hockings 2021, and Kibale National Park in Uganda: Cibot et al. 2015; Krief et al. 2020) and unprotected areas (e.g. Bossou in Guinea: Hockings, Anderson, and Matsuzawa 2006; Lawana in Sierra Leone: Garriga et al. 2019; and Bulindi in Uganda: McLennan and Asiimwe 2016). Chimpanzees cross roads to access dispersed food resources within their home ranges (Reynolds, Wallis, and Kyamanywa 2003; Hockings, Anderson, and Matsuzawa 2006; Cibot et al. 2015) or when females migrate between groups (McLennan et al. 2021). However, road‐crossing behavior in chimpanzees has been analyzed at two study sites only, Bossou (Hockings, Anderson, and Matsuzawa 2006; Hockings 2011) and Sebitoli (Cibot et al. 2015), with both studies based on around 30 direct observations of crossings. These studies showed that chimpanzees perceive roads as dangerous and can adapt their behavior to reduce the risks posed by road users (including vehicle traffic and pedestrians), for example by looking right and left before and during crossing, having adult males occupy the riskiest positions, paying attention to conspecifics, and waiting before crossing (Cibot et al. 2015).

In western Uganda, current infrastructural development including road upgrades (Uganda National Roads Authority 2023) pose a threat to many wild chimpanzee communities, especially those living outside protected areas (McLennan et al. 2021). Here, we focus on the chimpanzees in Bulindi, Hoima District. The Bulindi chimpanzee community provides an opportunity to investigate potential resilience of chimpanzees to anthropogenic landscape change, as it has lived for decades in an unprotected and highly human‐impacted area (McLennan and Hill 2012). Their home range is a mosaic of riverine forest fragments, croplands, village homes, and trading centers, bisected by a busy two‐lanes‐wide road linking two major urban centers. The chimpanzees have crossed this road for generations, despite increasing traffic in recent years (McLennan and Asiimwe 2016). This road poses a significant danger to chimpanzees and other wildlife, especially since it was upgraded in 2018 (widened and tarmacked), allowing vehicles to travel faster than before.

Our aim in this study was to analyze the choices and behavioral flexibility of wild chimpanzees when faced with the risk of crossing a busy main road, and to determine the factors that influence their road‐crossing behavior as a group or individually, using a 3‐year database of video material. We tested the following hypotheses:

**Hypothesis 1—Age‐sex influences on crossing behavior**: road crossing behavior varies according to sex and age class. Specifically, as described in chacma baboons (*Papio ursinus*; Rhine and Tilson 1987) and Bossou and Sebitoli chimpanzees (Hockings, Anderson, and Matsuzawa 2006; Cibot et al. 2015), we assume that mature males are most likely to take up forward and rearward positions (considered the riskier positions in previous studies), especially when the proportion of vulnerable individuals (immatures, mothers with dependents, injured individuals) in the crossing group increases (1.1). Similarly, mature males should be most likely to display cooperation and attention towards more vulnerable individuals during road crossings, such as waiting for conspecifics (Cibot et al. 2015) or exhibiting reassuring or protective behavior towards them, to enhance group protection (1.2). Generally, mature male chimpanzees are described as being bolder (Hockings 2007; Wilson, Hauser, and Wrangham 2007; Bertolani and Boesch 2008; McLennan and Hill 2010; Satsias et al. 2022). Consequently, females and immatures should display more vigilance than mature males by road crossing less frequently, waiting longer before crossing, crossing at a faster gait, and showing more apprehensive and cautious behaviors [e.g. traffic checking, avoidance; (1.3)].

However, because females spend more time with males when they are sexually receptive, i.e. when they display swellings of their anogenital region (Matsumoto‐Oda 1999; Pepper, Mitani, and Watts 1999), we expect their behavior to vary during their sexual cycle, and we hypothesize that females with anogenital swellings should behave in a similar way to males, i.e. less vigilantly (1.4).

**Hypothesis 2—Chimpanzee risk assessment**: because chimpanzees can assess the risk of road crossings (Hockings 2011; Cibot et al. 2015), they adjust the way they cross to the degree of risk involved. Specifically, we predict that they should choose to cross when there is less traffic (2.1) and at safer crossing points, i.e. road sections with good visibility (low gradient and no bend), high vegetal coverage on at least one side of the road (allowing chimpanzees to hide before or disappear from view quickly after crossing), and reduced vehicle speeds [due to the presence of speed humps; (2.2)]. Additionally, as the level of risk presented by the road increases (higher traffic intensity, reduced visibility of oncoming traffic and detectability of chimpanzees; Hockings, Anderson, and Matsuzawa 2006), chimpanzees are expected to be more vigilant (2.3), i.e. individual waiting times before crossing should increase, chimpanzees should cross faster and show more cautious behaviors.

**Hypothesis 3—Social influences on crossing behavior**: chimpanzee behavior during road crossings varies with the size and composition of the crossing group (Cibot et al. 2015). Larger groups may take more time to organize themselves in potentially risky situations, such as crossing a road (Hockings 2011). Therefore, we hypothesize that as group size increases, waiting time before crossing will also increase (3.1). Furthermore, humans cross roads more rapidly in small groups than when many people cross (Gates et al. 2006; Peters et al. 2015; Onelcin and Alver 2017). Thus, we expect the chimpanzees to cross faster in smaller groups to limit their exposure time on the road (3.2). We also predict that they will show more cautiousness in smaller crossing groups (3.3).

## 2 Methods

### 2.1 Ethical Note

These observations of wild chimpanzees were noninvasive; observers strived to maintain a minimum distance from the chimpanzees of 7 m and avoided interacting with them. All national and international guidelines were followed. This research adhered strictly to ethics guidelines detailed by the Association for the Study of Animal Behaviour (UK) and the American Society of Primatologists Principles for the Ethical Treatment of Nonhuman Primates. The study was approved by the Uganda Wildlife Authority and the Uganda National Council for Science and Technology.

### 2.2 Study Site and Subjects

**Figure 1:**
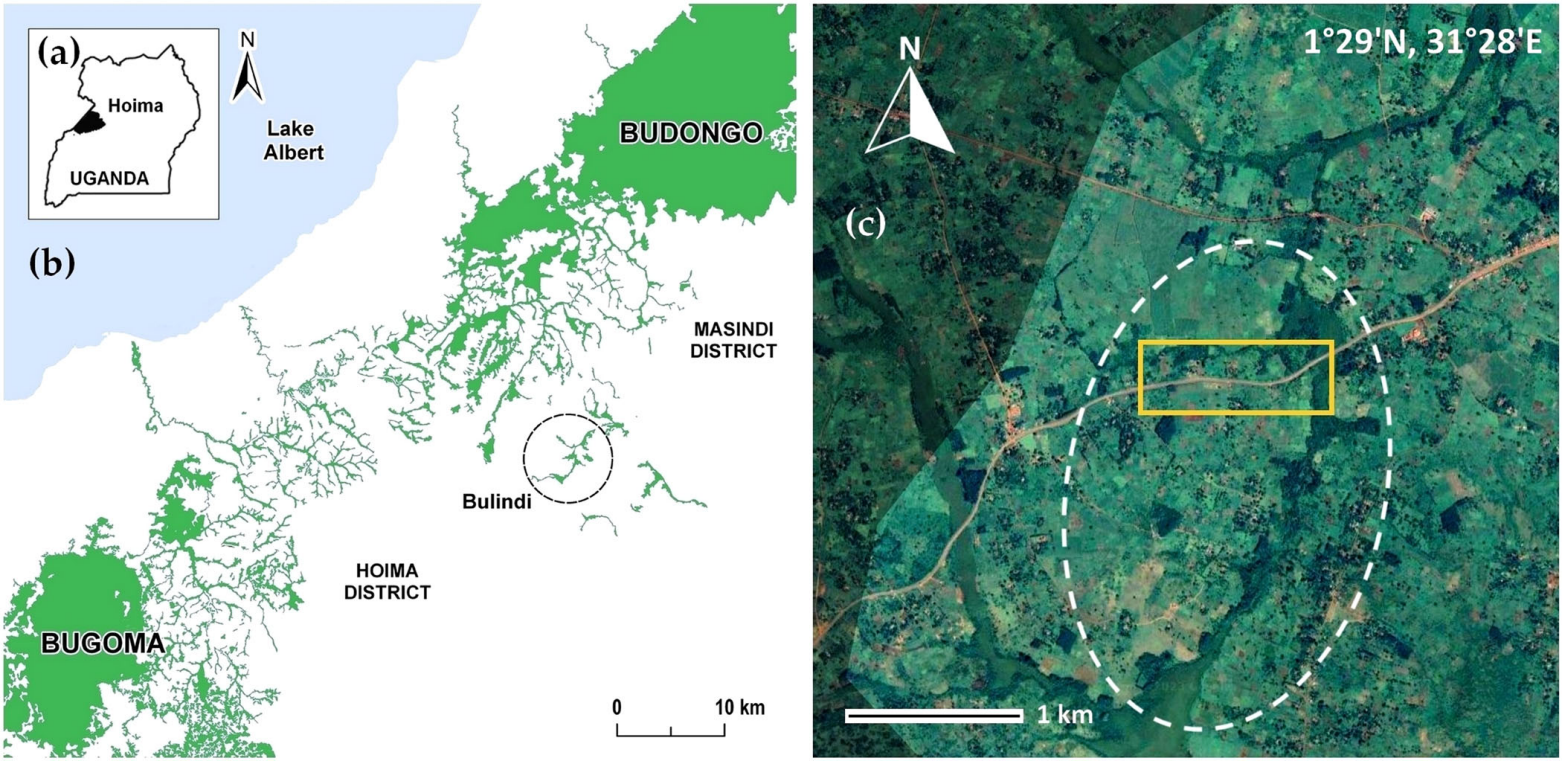
Location of Bulindi, Hoima District. (a) Map of Uganda showing the location of Hoima District in western Uganda. (b) Map (adapted from Cibot et al. 2019) showing the location of the Bulindi study site (encircled), midway between the Budongo and Bugoma forests in the north and south, respectively. (c) Satellite map of the Bulindi area (Bulindi: 1°29′ N, 31°28′ E, Hoima‐Masindi Road) (map adapted from Google Earth). The chimpanzees' 20 km^2^ home range consists of small fragments of riverine forest and papyrus swamps (visible in dark green on the map) that are surrounded by crop fields, exotic wood plantations, homes and village areas. The core of their home range is represented by the white dashed oval (ca. 4 km^2^). It is bisected by a major road (visible at the center) that connects Hoima city to Masindi town. Numerous secondary roads and village paths also run through the area. The portion of road crossed by chimpanzees during this study is framed in yellow.

Bulindi (1°28′N, 31°28′E) is located in Hoima District, western Uganda, midway between the Budongo and Bugoma Central Forest Reserves (Figure 1). Since the 1990s, most forest outside the reserves has been converted to farmland (McLennan 2008; Mwavu and Witkowski 2008), and the human population has grown rapidly (156 persons per km^2^ in 2014; Uganda Bureau of Statistics 2016). However, more than 260 chimpanzees comprising 10 or more communities survive in fast‐changing human‐dominated habitats outside the reserves, including the Bulindi chimpanzee community (McLennan 2008; McCarthy et al. 2015).

Chimpanzees at Bulindi were first studied in 2006–2008, when the community comprised about 30 unhabituated individuals (McLennan and Hill 2010). Research has been ongoing since 2012 and the chimpanzees were habituated by 2015 (McLennan et al. 2019). The present study covered the period from November 2018 to December 2021 (38 months). During this period, community size varied from 20 to 22 individuals (Table 1). We estimated the age class of individuals born before 2012 from body size and other physical and behavioral characteristics. At the start of the study, the community comprised 20 individuals including 3 adult males (≥ 12 years old), 5 adult females (≥ 12 years old or at first birth, if earlier), 4 sub‐adults or adolescents (≥ 8 years old), 4 juveniles (≥ 4 years old) and 4 infants (newborn to 3 years old) (Sugiyama 2004). Some individuals transitioned to an older age class during the study (e.g. 3 juveniles entered adolescence; 3 subadult males became young adults; Supporting Information [SI]**:** Table S1). At the end of the study, community size was 22 individuals including 5 adult males, 6 adult females, 3 sub‐adults, 4 juveniles and 4 infants. The alpha male at the start of the study (SL) disappeared after December 2019 and was replaced by MO, who was alpha male during the remainder of our study. For behavioral analyses, we chose to lump late‐subadults (from 10 years old) with adults, hereafter referred to as “mature individuals”, and young sub‐adults (8–9 years old) with juveniles, hereafter referred to as “immature individuals”. Socially and behaviorally, late subadult males at Bulindi are prominent in social networks (Satsias et al. 2022), range independently of their mothers, and frequently lead travel parties, in the same way as adult males. This is not the case with young subadults. Table 1 provides information about the composition of the population during the study period.

**Table 1:** Number of individuals in each age‐sex class in the Bulindi chimpanzee community and community size per year (calculated at year‐end) during the study period.

YEAR	2018^a^	2019	2020	2021
MATURE MALES (10+)	**3**	**6**	**5**	**5**
MATURE FEMALES (10+)	**5**	**5**	**6**	**6**
IMMATURES (4+)	**8**	**5**	**7**	**7**
INFANTS (0–3)	**4**	**6**	**3**	**4**
COMMUNITY SIZE	20	22	21	22

^a^ Two subadult males who were classed as ‘mature’ from 2019, featured in a single video in November 2018 when they were still ‘immature’ according to our definition (≤ 9 years old). For convenience, we treated them as mature in that video clip.

**Figure 2:**
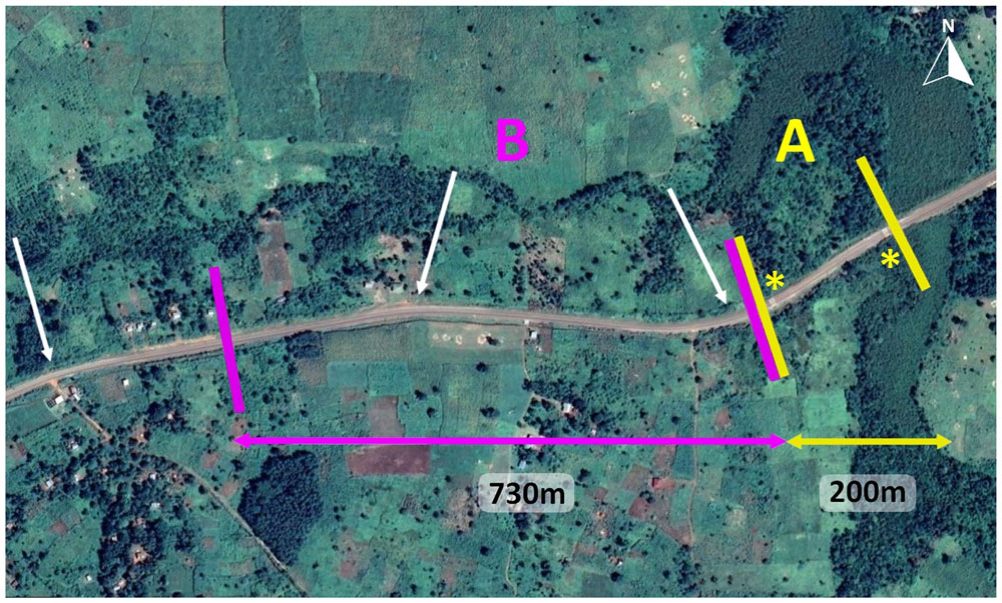
Map showing the 1 km stretch of road where Bulindi's chimpanzees habitually cross. Zone A is delimited by yellow lines and zone B by pink lines. Two speed humps, 180 meters apart, are marked with yellow asterisks. Notable bends affecting visibility are indicated by white arrows. Dark green patches to the north of the road and on either side of the road at the far right of the map are fragments of riverine forest, regenerating forest, and papyrus swamp (map adapted from Google Earth).

Between 2006 and 2014, about 80% of forest in Bulindi was cleared entirely for farming (McLennan et al. 2020), shrinking main forest fragments used by the chimpanzees from ca. 122 to 26 ha and leading to an increase in their use of farmland for traveling and foraging. Remaining forest occurs patchily on either side of a 10.5 m‐wide major road (Figure 1) that connects Hoima city and Masindi town—two main urban centers with over 110,000 inhabitants each (Uganda Bureau of Statistics 2016). This road, which was widened and tarmacked in 2018 in response to infrastructure needs including oil sector development occurring regionally (Mawejje 2018; Ogwang and Vanclay 2019; McLennan et al. 2021), receives high volumes of motor vehicles (motorbikes, cars, taxis, buses, trucks, tourist vehicles) along with cyclists and pedestrians. Living in this highly human occupied area, Bulindi's chimpanzees are confronted with the frequent need to cross this busy road to access food sources located on either side (McLennan and Asiimwe 2016). One fatality involving an adult female which collided with a taxi vehicle prior to the upgrading of the road has already been reported (McLennan and Asiimwe 2016). During our study, two speed humps were installed by the road authorities on the section of the road where this female died and where the chimpanzees often crossed (Figure 2) to slow down vehicles and reduce the risk of accidents. These humps were under construction from late‐2018, including use of temporary humps made from sand, which necessitated vehicles to slow down; the permanent, single cement humps were completed by 2020.

### 2.3 Data Collection and Analysis

#### 2.3.1 Fieldwork: Data Collection

Road crossing observations took place on a 1‐km stretch of the Hoima‐Masindi road. We divided the road into two sections which we perceived presented the chimpanzees with different levels of danger (Figure 2):

– Zone A (in yellow): 200 m long; boundaries are the speed humps at the west and east sides. There is a road bend just beyond the western hump. We considered this section as a comparatively “safe” zone because traffic speed is limited by the two speed humps positioned 180 meters apart. The vegetation cover is dense on the northern side of the road (regenerating forest), and medium on the southern side (fallow fields and bushes), providing cover for the chimpanzees prior to and after crossings.
– Zone B (in pink): 730 m long; boundaries are the road bend and speed hump on the eastern side (separating zones B and A). On the western side, the boundary was set at the most westerly point where chimpanzees were observed crossing. We considered this section as a “high‐risk” zone because, while vehicles are slowed down by the hump on the east side, they can accelerate quickly and vehicles can travel at high speed on the remaining section. Moreover, two road bends affect the visibility of oncoming traffic in this section (Figure 2). There is medium (bushes) to low vegetation cover (open croplands and gardens) on both sides of this section, making it difficult for chimpanzees to approach the road surreptitiously or stay hidden by the roadside.

During the study period, the chimpanzees were followed 5 to 7 days per week by field assistants JB and TS. Both assistants were present during morning hours, while only JB worked during afternoon and evening hours. Field assistants recorded party (subgroup) composition and activity every 30 min (party scans; see Satsias et al. 2022) and noted the time of road crossings when these occurred between scans. Road crossings were filmed opportunistically by the field assistants using a digital camera (Table S3), sometimes with an alternate view filmed by MRM. When a crossing seemed imminent (e.g. as indicated by the direction of chimpanzees' travel towards the road), where possible field assistants would try to get ahead of the chimpanzees and film the crossing, usually by waiting at the road near to where the chimpanzees seemed most likely to cross. Sometimes the field assistants would actively intervene to encourage drivers to slow down or advise pedestrians to wait so that the chimpanzees could cross safely. Some videos did not capture the entire crossing, as the assistants could not always reach the road before a crossing began or because individuals crossed at different locations on the road. These videos, representing 22 separate road crossings, were not retained in the dataset for behavioral analysis. For these reasons, and due to the fission‐fusion social organization of chimpanzees which meant the field assistants normally followed only a portion of the community, many crossings were undoubtedly missed. Crossings were recorded no earlier than 0700 h and no later than 1900 h, corresponding to field assistants' working hours, as well as the times chimpanzees typically leave their nests in the morning and build their nests in the evening. Additionally, the chimpanzees were not normally followed from 1200 to 1400 h, and sometimes not relocated until 1430 h or later. Thus, crossings during early afternoons were rarely observed. However, it is worth noting that Bulindi's chimpanzees are unlikely to cross the road in the early afternoon as they usually rest in undergrowth from late morning to about 1600 h (unpublished data). Overall, our dataset represents a sample of crossings that occurred during the study period; while these were recorded opportunistically, we believe they are sufficiently representative of the chimpanzees' road crossing behavior.

#### 2.3.2 Video Analysis and Behavioral Observations

Behavioral data collection from the videos was carried out using the Behavioral Observation Research Interactive Software (BORIS, v. 7.11.1). All behaviors and descriptions considered in this study are listed in the ethogram in Table S2.

A “road‐crossing event” was defined as the observation of at least one chimpanzee crossing the road (Cibot et al. 2015). “Total crossing‐group size” was the total number of individuals excluding infants that crossed during a road‐crossing event. It was considered a “single crossing‐event” when two (or more) subgroups of chimpanzees crossed the road in the same direction, on the same stretch of road, within 30 min (modified from Cibot et al. 2015). A chimpanzee was considered to be in the same subgroup as the preceding individual if they crossed the road within 1 min of each other at the same location. Hereafter, we will refer to the crossing group as a “(sub)group”, depending on whether the chimpanzees crossed as a single group or in two or more sub‐groups. The total duration of a crossing event—the “crossing duration”—extends from the moment the first chimpanzee (or leader) steps on to the asphalt to the moment the last individual to cross leaves the asphalt. The “individual waiting time” was the elapsed time between the start of the crossing by the first chimpanzee in the (sub)group, and the start of a subsequent individual's crossing (modified from Cibot et al. 2015). Leaders were therefore excluded from this analysis.

For each crossing event we noted the date and time; location (i.e. zone A or B); direction and extent of cover on both sides of the road (i.e. low vegetation cover is open croplands or gardens; medium cover is fallow fields or bushes; dense cover is regenerating forest); any interventions by the field assistants (i.e. vocalizing or gesturing to road users, or adopting a guarding posture on the road); the total crossing‐group size (excluding infants, i.e. all individuals visible in the video that were ≥ 4 years old at the end of the video year, the age that first independent road crossings of young chimpanzees are observed at Bulindi) and subgroup size when the chimpanzees did not cross all together; the composition of the total crossing‐group and subgroups; and the order in which group members crossed. To gain a measure of the intensity of motor and non‐motor traffic at the time chimpanzees chose to cross the road, we counted the number of motor vehicles (motorbikes, cars, taxis, buses, trucks) and non‐motor road users (cyclists and pedestrians) visible per minute during recordings (i.e. when chimpanzees had approached the road and a crossing was imminent). Road users that were clearly audible (i.e. close to the camera and/or interacting with field assistants) but not visible in the camera field were also taken into account, even though the type of vehicle or the number of pedestrians could not always be identified (the default value was 1). These values were minimum estimates because we could not assess all traffic out of the camera range. Time of crossing and composition of (sub)groups were corroborated by the field assistants' scan data and written notes. Each crossing individual was assigned an age‐sex class (mature female, mature male, or immature individual under 10 years old). The anogenital (estrus) swelling status of each mature female was scored daily for the duration of the study: “0” = no swelling, “1” = partially swollen and “2” = maximally swollen (Satsias et al. 2022).

An individual's behavior was analyzed from the moment they appeared in the video (or from the beginning of the video if they were already visible) until the moment they disappeared from the camera field, or if applicable until the end of the recording. For directed behaviors involving another individual, the identity of the receiver or emitter was also noted. Individual waiting times could not be calculated for all videos and subjects because the roadside where individuals crossed from was not always visible in the camera field. However, the dataset of usable videos was sufficient to conduct a meaningful analysis of waiting times (*N* = 66 videos representing 416 individual waiting times).

We defined two crossing gaits according to Cibot et al. (2015): fast gait (running, including intermediate speeds such as “ambling,” defined by Schmitt et al. [2006] as a fast walking gait) or slow gait (walking). Crossing gait was scored for each individual, and we noted any lameness caused by temporary injuries. For dependents (i.e. infants and young juveniles), we noted if they were carried by their mother for at least part of the crossing. We defined “vulnerable individuals” as immatures (slower, less experienced, less visible to drivers), females with dependents (slower, vigilance can be affected by the dependent) and injured individuals (individuals with temporary injuries that cause them to limp during locomotion and cross slower). We further identified the following behavioral categories (Table S2): protective and cooperative behaviors, such as reassurance gestures, individuals adopting guarding postures or waiting for conspecifics (excluding dependents waiting for their mother); apprehensive and cautious behaviors, such as traffic checking, retreating or avoidance; and reckless behaviors, such as stopping in the middle of the road or charging displays performed during the crossing.

#### 2.3.3 Statistical Analysis

##### 2.3.3.1 Evaluating the Extrinsic Factors

We first compared traffic intensity during crossings (i.e. the number of motor vehicles and non‐motor road users per minute) in each video recording according to the time of day (5 time slots of 2 h between 0700 and 1900 h) to assess if traffic intensity during chimpanzee crossings varied significantly over the course of the day.

##### 2.3.3.2 Testing Our Hypotheses

According to *Hypothesis 1—Age‐sex influences on crossing behavior*, we expect variation in road crossing behavior according to sex and age class. Overall, we expect that females and immatures will display more vigilance than mature males; hence, they should cross the road less frequently than mature males. We compared the rate of taking part in road crossing per crossing event for each category of individual (immatures, mature females, mature males), to test whether any category crossed more frequently than others. Because these data were not normally distributed (using Shapiro‐Wilk normality test), we used analysis of variance (ANOVA) with permutation tests (non‐parametric ANOVA). If the ANOVA was significant, we applied post hoc permutation tests for independent samples and corrected the p‐values (*P’*) with the Bonferroni correction (to avoid type I error, i.e. false‐positives). We used the *aovp* function in the *lmPerm* package (Wheeler and Torchiano 2016) for the permutation tests.

As we differentiated the two crossing zones according to level of danger, we compared the observed age‐sex composition of crossing groups in each zone with the expected theoretical proportion (i.e. mean proportion over the study period of each age‐sex category in the total group) using Chi‐square tests. In the same way, we compared crossing rates of females in different anogenital swelling stages between the two zones, as we expect their behavior to vary during their sexual cycle. Specifically, we expect females to cross in a similar way to males, i.e. less vigilantly, when maximally swollen.

Furthermore, we tested our different hypotheses by fitting generalized linear mixed models (GLMM) using the *glmer* function in the R package *lme4* (Bates et al. 2015). Several models were fitted including alternatively, as the dependent variable, an individual's position [binary outcome: being in a risky position (1) or in a protected position (0)], protective behavior [binary outcome: showing protective behaviors (1) or not (0)], crossing gait [binary outcome: traveling fast (1) or not (0)], cautious behavior [binary outcome: showing apprehensive/cautious behaviors (1) or not (0)] and waiting time (continuous variable; note that waiting time varies from 1 s to 7 min, so we log‐transformed this variable to ensure normality and homoscedasticity assumptions). We chose the Binomial or the Gaussian family depending on the nature of the dependent variable.

In general, we expect that mature females and immatures will wait longer before crossing, travel at a faster gait to limit their exposure time on the road, and exhibit more cautious behaviors. Individual chimpanzees and crossing events were both added as random effects to take into account possible correlations amongst observations of the same individual and amongst observations of individuals in the same crossing event (pseudo‐replication). We compared the fit of the full model with that of the null model comprising only the random effects using the Akaike Information Criteria (AIC; Onyango 2009) coupled to an ANOVA. In addition, we expect that mature males will adopt riskier positions during crossings (i.e. forward or rearward positions) than other age‐sex categories (fixed effect), and should display more protective behaviors towards vulnerable individuals (immatures, mothers with dependents, injured individuals; fixed effect), especially when the proportion of vulnerable individuals in the crossing group increases. We thus included an interaction between age‐sex category and the proportion of vulnerable individuals. Here, we compared the fit of the full model, including age‐sex category and the interaction with the proportion of vulnerable individuals, with that of the null model comprising only the fixed control effect (age‐sex category) and random effects. We also tested the influence of anogenital swelling status (i.e. ‘0’ = no swelling, ‘1’ = partially swollen and ‘2’ = maximally swollen) on the crossing behavior of females, and the effect of carrying a dependent. Additionally, we examined the effect of field assistant interventions on chimpanzees' cautious behavior.

According to *Hypothesis 2—Chimpanzee risk assessment*, we expect that chimpanzees adjust how they cross to the degree of risk involved. We expect that traffic intensity during crossings and crossing zone (fixed effects) will have a significant influence on individual waiting times (after the first chimpanzee to cross), as well as on crossing gaits and cautiousness of individuals. The individual's age‐sex category was included as a fixed control factor. Individual chimpanzees and crossing events were added as random effects when testing the influence of traffic intensity, whereas only individual chimpanzees were added as random effects when testing the crossing zone because crossing events are nested within the crossing zone (i.e. crossing events always occurred in one zone only). The extrinsic predictors (traffic intensity or crossing zone) were tested on each dependent variable, i.e. an individual's waiting time, crossing gait and cautious behavior. We compared the fit of the full models with those of the null model comprising the fixed control effect (age‐sex category) and random effects.

According to *Hypothesis 3—Social influences on crossing behavior*, we expect that chimpanzee behavior during road crossings varies with the size and composition of the crossing group. We expect that (sub)group size (fixed effect) and the proportion of mature males (fixed effect) will influence an individual's waiting time, crossing gait and cautiousness. Age‐sex category was included as a fixed control factor and individuals were added as random effects. The crossing event was not included as a random effect as the fixed effects do not vary within crossing events. We compared the fit of the full model [including (sub)group size or proportion of mature males] with that of the null model comprising the fixed control effect (age‐sex category) and the random effect.

All statistical procedures were performed using R (v.4.0.3; R Core Team 2022) and the significance threshold was set at *p* < 0.05.

## 3 Results

The dataset comprised a total of 176 videos of chimpanzees crossing the road, recorded on 111 different days and representing 129 distinct road‐crossing events. Among these events, we recorded 190 crossing (sub)groups. The dataset represents 6.25 h of video material and the duration of individual videos ranges from 14 s to 16 min with an average duration of 2.1 ± 2.4 (mean ± SD) minutes.

Over the 38‐month study, we made video recordings of chimpanzees crossing the road an average of 4.2 ± 3.0 (range 0–12 crossing events) times per month. Road crossings were recorded mainly in morning hours between 0700 and 0900 (33.3% = 43/129) and in the evening between 1700 and 1900 (31.8% = 41/129). No recordings were made between 1300 and 1500 h.

### 3.1 Extrinsic Factor: Traffic Intensity During Crossings

**Figure 3:**
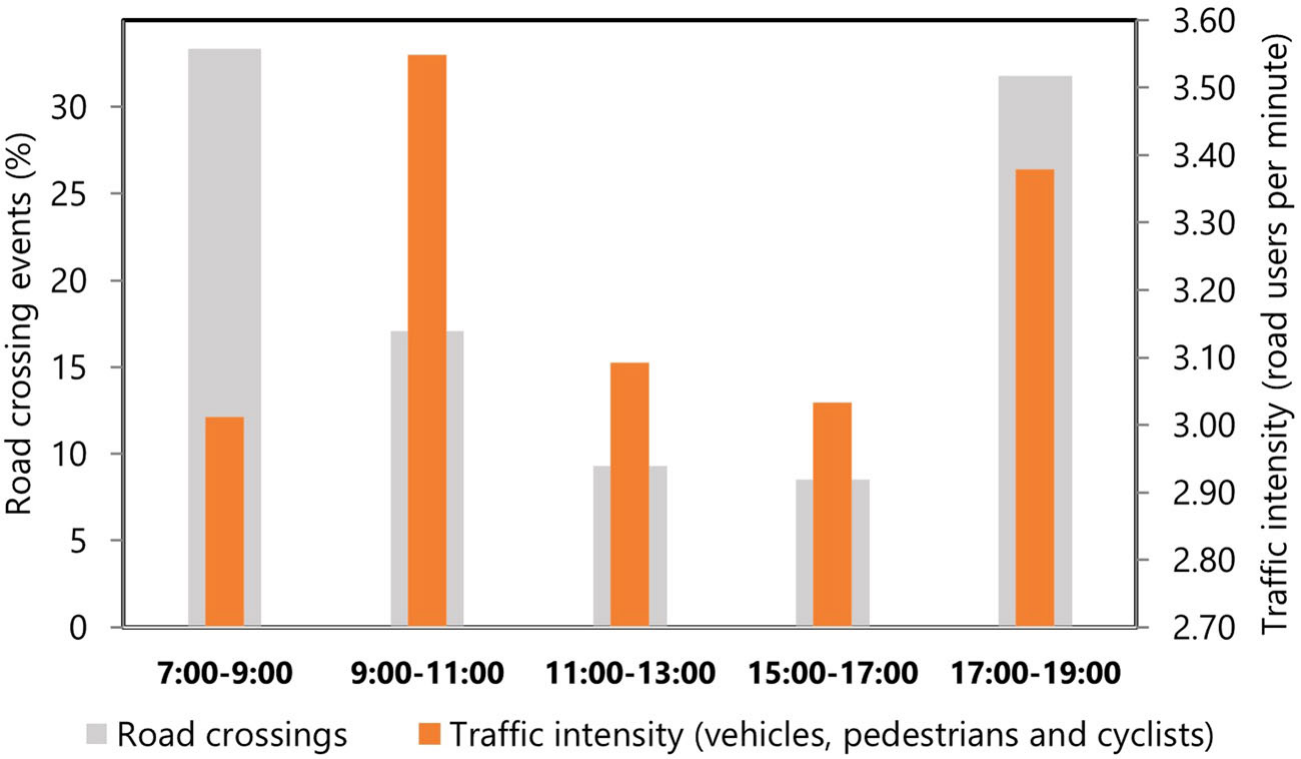
Barplots showing the percentage of road crossing events in relation to traffic intensity (the average number of road users [motorized vehicles, cyclists, and pedestrians] per minute during crossings) at different times of day. Sample size of crossing events (*N* = 129): 7:00–9:00 = 43 events; 9:00–11:00 = 22; 11:00–13:00 = 12; 15:00–17:00 = 11; 17:00–19:00 = 41). No crossing events were observed between 13:00 and 15:00 h, thus information on traffic intensity was not available.

Mean traffic intensity per minute during crossings was 0.8 ± 1.4 (range 0–13.2) pedestrians, 0.2 ± 0.6 (range 0–5.1) cyclists, and 2.4 ± 1.8 (range 0–12.7) motor vehicles, with motorbikes being the most frequent vehicles representing 63.2% (592/936) of motor road users. Traffic intensity during crossings was highest between 9:00–11:00 and 17:00–19:00 but did not differ significantly depending on the time of day (nonparametric ANOVA: number of motor vehicles per minute: *F*_4_ = 0.378, *p* = 0.824; number of pedestrians and cyclists per minute: *F*_4_ = 0.820, *p* = 0.514; Figure 3). Most crossings occurred between 7:00 and 9:00 when traffic intensity was relatively low and 17:00–19:00 when it was relatively high (Figure 3). Thus, chimpanzees did not appear to adjust the time of their road crossings according to traffic intensity, but instead selected early morning and late afternoon and evening hours to cross.

### 3.2 Overall Crossing Behavior

Overall, 34.9% of the mature males, 24.8% of mature females and 27.4% of immatures in the community crossed the road per event recorded. Mature males were recorded crossing significantly more often than females (Permutation test: *F*_1_ = 12.72, *P’* = 0.001) and immature chimpanzees (Permutation test: *F*_1_ = 6.317, *P’* = 0.025). Overall, the chimpanzees crossed preferentially in the safer zone A compared to the riskier zone B. Of 129 crossing events in the dataset, 49 took place in zone B (38.0%), which is 3.7 times longer in length than zone A (see Figure 2), and 80 crossing events took place in zone A (62.0%) (*χ* ^2^ test for given probabilities: *χ* ^2^_2_ = 7.450, *p* = 0.006). Individuals waited an average of 39.4 (range 0.1–421.4) seconds before crossing after the (sub)group leader. We observed an average of 8.9 ± 7.3 cautious behaviors per crossing event, primarily consisting of traffic checking (1103/1143 occurrences; 96.5%). Remaining cautious behaviors comprised avoidance and retreat (40/1143 occurrences; 3.5%).

Over the study period, the average proportion of individuals in the different age‐sex classes was as follows (excluding infants): 29.9% were mature males, 33.3% were mature females and 36.8% were immatures. The observed distribution of these categories when crossing in zone B was 59.5% for males, 17.2% for females, and 23.3% for immatures. This distribution differed significantly from the average distribution of age‐sex categories in the community (*χ* ^2^ test for given probabilities: *χ* ^2^_2_ = 94.37, *p* < 0.0001), while the observed distribution of these age‐sex categories when crossings in zone A did not differ from the community average (29.5% for males, 33.2% for females and 37.3% for immatures; *χ* ^2^ test for given probabilities: *χ* ^2^_2_ = 0.067, *p* = 0.967). Thus, mature males were more likely to cross in the riskier zone B than females and immatures.

A summary of the following results from the GLMM analyses is shown in Table 2.

**Table 2:** Summary of parameter estimates for the significant models (GLMM).

Parameter	Estimate	SE	*Z* value or *t* value	*p* value	AIC full vs null	*p* value full vs null
Hypothesis 1: Age‐sex influences on crossing behavior						
Crossing gait ~ age‐sex (AS) category					987 vs 1003	< 0.0001
(Intercept)	1.76	0.37	4.77	< 0.0001		
Mature female	−1.32	0.52	−2.53	0.0114		
Mature male	−3.08	0.53	−5.79	< 0.0001		
Cautious behavior ~ AS category					857 vs 875	< 0.0001
(Intercept)	0.93	0.22	4.26	< 0.0001		
Mature female	0.94	0.27	3.54	< 0.0001		
Mature male	1.66	0.29	5.67	< 0.0001		
Individual waiting time ~ AS category					1315 vs 1320	0.012
(Intercept)	2.44	0.17	14.07	< 0.0001		
Mature female	−0.0005	0.15	−0.003	0.99		
Mature male	0.47	0.16	2.97	0.0103		
Position ~ AS category					547 vs 562	< 0.0001
(Intercept)	−2.10	0.26	−8.23	< 0.0001		
Mature female	1.24	0.33	3.74	0.0002		
Mature male	1.76	0.35	5.04	< 0.0001		
Protective behavior ~ AS category					515 vs 558	< 0.0001
(Intercept)	−1.33	0.19	−6.98	< 0.0001		
Mature male	0.74	0.25	3.01	0.0026		
Immature	−1.17	0.33	−3.57	0.0004		
Hypothesis 2: Chimpanzee risk assessment						
Crossing gait ~ AS category + traffic intensity					920 vs 987	< 0.0001
(Intercept)	0.77	0.41	1.87	0.06		
Mature female	−1.48	0.56	−2.66	0.008		
Mature male	−3.44	0.57	−6.04	< 0.0001		
Motor traffic	0.49	0.07	7.25	< 0.0001		
Non‐motor traffic	0.26	0.11	2.44	0.015		
Cautious behavior ~ AS category + crossing zone					922 vs 926	0.021
(Intercept)	0.80	0.16	5.03	< 0.0001		
Mature female	0.74	0.24	3.11	0.002		
Mature male	1.37	0.25	5.42	< 0.0001		
Crossing zone	−0.46	0.20	−2.33	0.020		
Individual waiting time ~ AS category + crossing zone					1476 vs 1482	0.005
(Intercept)	2.72	0.12	21.81	< 0.0001		
Mature female	0.05	0.18	0.27	0.79		
Mature male	0.58	0.19	3.07	0.009		
Crossing zone	−0.47	0.17	−2.82	0.005		
Hypothesis 3: Social influences on crossing behavior						
Crossing gait ~ AS category + (sub)group size					985 vs 987	0.04
(Intercept)	2.16	0.43	5.08	< 0.0001		
Mature female	−1.35	0.53	−2.52	0.012		
Mature male	−3.21	0.55	−5.88	< 0.0001		
(Sub)group size	−0.04	0.02	−2.06	0.04		
Individual waiting time ~ AS category + (sub)group size					1468 vs 1482	< 0.0001
(Intercept)	1.77	0.24	7.34	< 0.0001		
Mature female	0.07	0.17	0.41	0.69		
Mature male	0.60	0.17	3.49	0.004		
(Sub)group size	0.08	0.02	4.05	< 0.0001		

### 3.3 Hypothesis 1—Age‐Sex Influences on Crossing Behavior

**Figure 4:**
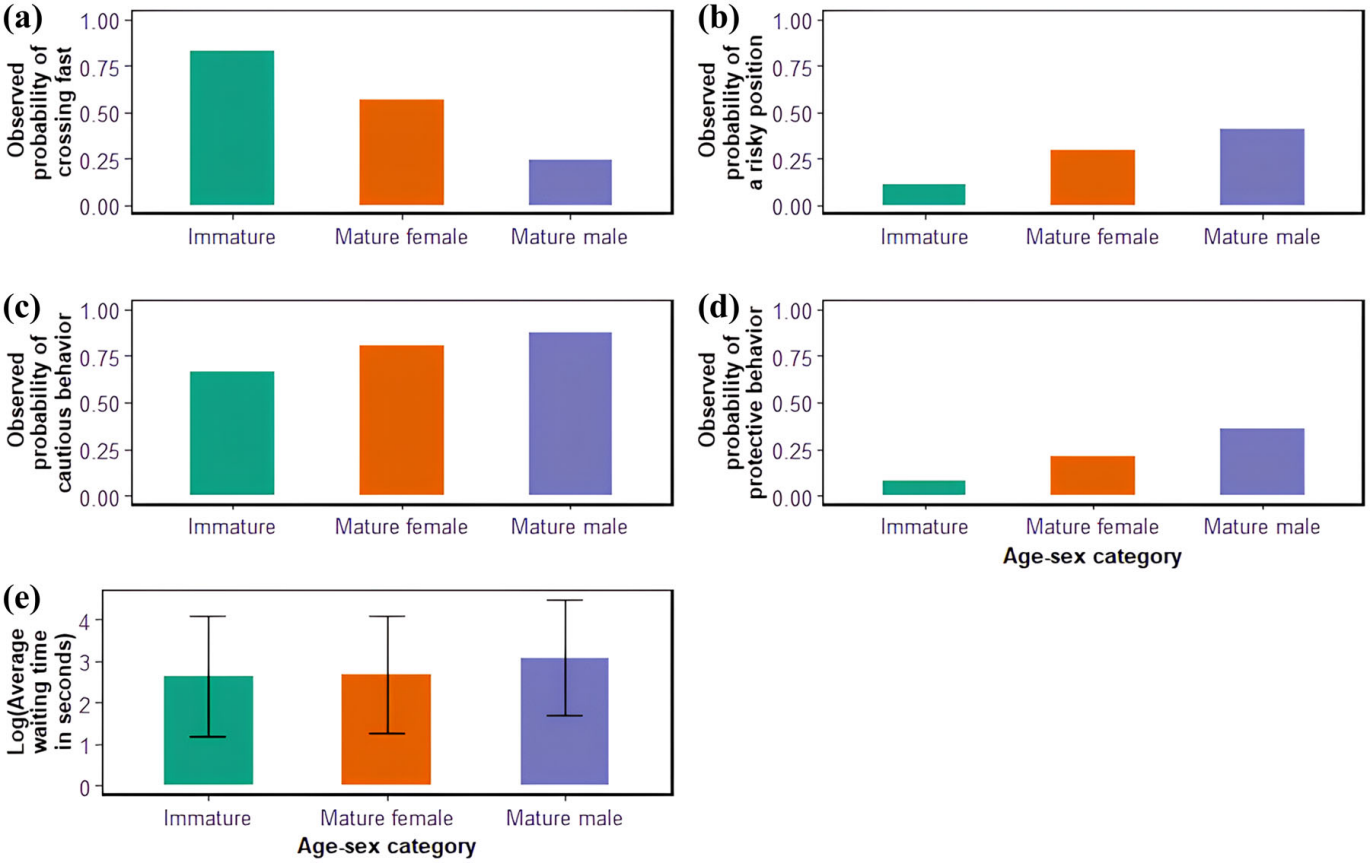
Barplots showing the observed probabilities of crossing at a fast gait [i.e. running or ambling; (a)], adopting a risky position [i.e. forward or rearward; (b)], exhibiting cautious behaviors (c) and exhibiting protective behaviors (d), and the log‐transformed average individual waiting times [in seconds; (e)], according to the different age‐sex categories; immatures in green, mature females in orange, mature males in purple (this color code remains the same throughout the article).

Overall, there was a general effect of age‐sex category on crossing gait, cautious behavior, and waiting time (null model comparison: *χ* ^2^ = 19.83, df = 2; *p* < 0.0001; *χ* ^2^ = 22.27, df = 2; *p* < 0.0001; *χ* ^2^ = 8.85, df = 2; *p* = 0.012; respectively). According to the models, the likelihood of a mature male crossing the road at a fast gait (running or ambling) was 21%, while it was 61% and 85% in mature females and immatures, respectively. Thus, mature males crossed at a slower pace compared to mature females and immatures. The likelihood of an individual showing cautious behavior was 67% in immatures, compared to 81% and 88% in mature females and mature males, respectively (Figure 4). The proportion of vulnerable individuals in the crossing (sub)group had no influence on cautious behavior (*χ* ^2^ = 2.13, df = 1; *p* = 0.14).

There was an effect of age‐sex category on an individual's position when crossing (i.e. risky or protected; null model comparison: *χ* ^2^ = 19.07, df = 2; *p* < 0.0001), but including the interaction with the proportion of vulnerable individuals in the crossing (sub)group had no influence (*χ* ^2^ = 5.65, df = 3; *p* = 0.13). According to the significant model, the likelihood of an individual crossing in a risky position was 11% in immatures, 30% in mature females, and 41% in mature males (Figure 4).

**Figure 5:**
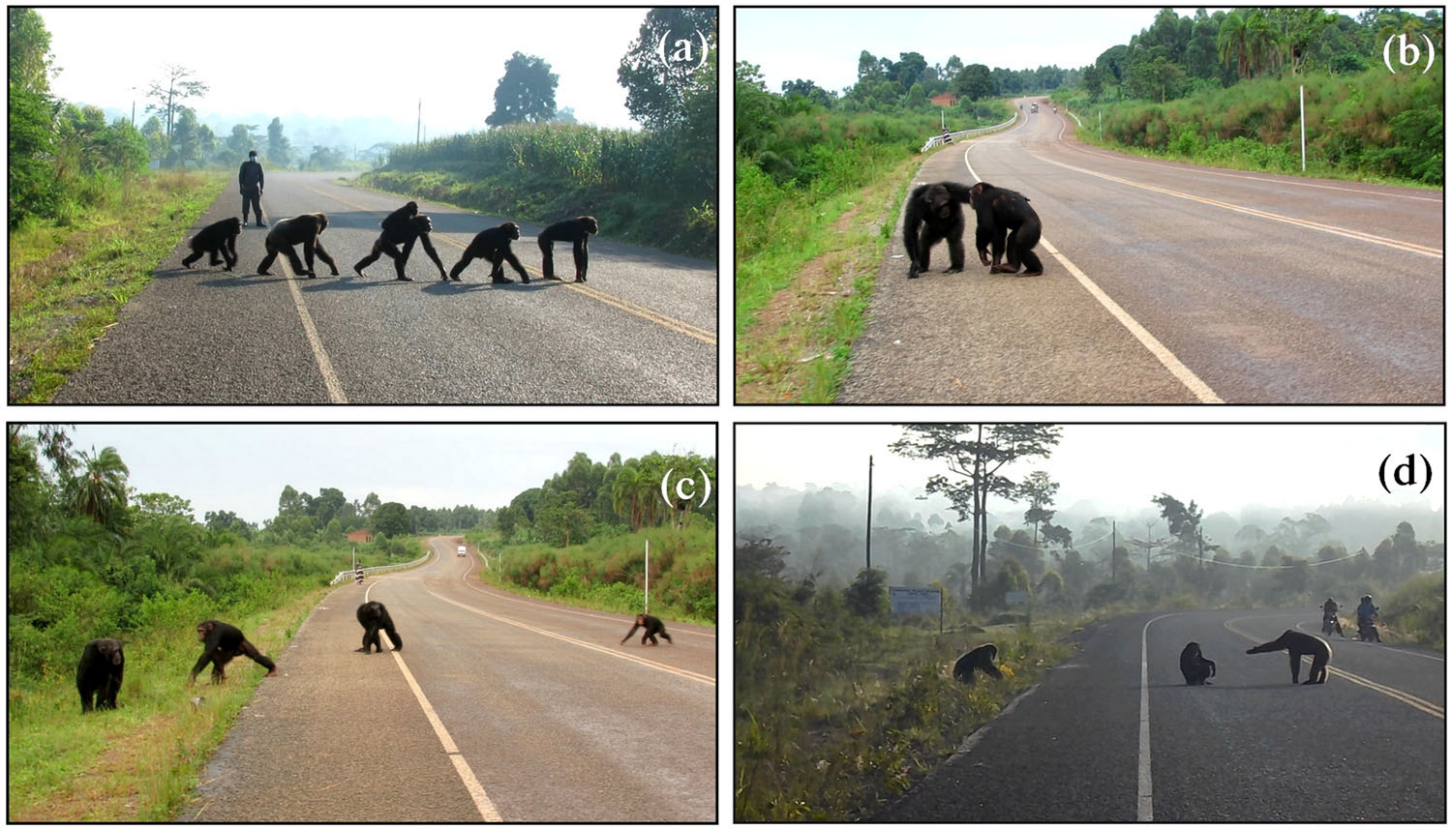
Video screenshots showing mature male chimpanzees exhibiting protective behaviors during road crossings. (a) JK (rightmost individual) showing guarding behavior, i.e. standing in a quadrupedal and alert posture on the road for more than 3 s without moving (Hockings 2011), while others cross. Field assistant TS is standing nearby while the chimpanzees cross. (b) MR making a reassurance gesture, i.e. gesture produced in reaction to recipient's distress or solicited from another (here, embracing), to GD who was limping from a temporary injury. (c) MR (leftmost individual) and MO (third individual from the left) waiting for immature MK (rightmost individual). (d) SL gesturing to MR (at the roadside on the left) to cross while JK sits on the road, watched by waiting motorcyclists.

We observed a significant effect of age‐sex category on protective behaviors (null model comparison: *χ* ^2^ = 47.18, df = 2; *p* < 0.0001), but the proportion of vulnerable individuals in the crossing (sub)group had no influence (*χ* ^2^ = 0.19, df = 3; *p* = 0.98). The likelihood of an individual exhibiting protective behaviors was 8% in immatures, 21% in mature females, and 36% in mature males (Figures 4 and 5). Mature males presented significantly more protective behaviors (estimate = 0.74, *z* value = 3.012, *p* = 0.003) while immatures showed significantly less protective behaviors (estimate = −1.17, *z* value = −3.568, *p* = 0.0004). However, there was also important interindividual variation; for example, among males, two individuals including MO, the alpha male for most of our study period, accounted for 67.9% [36/53] of occurrences of protective behavior.

### 3.4 Influence of Females' Anogenital Swelling Status

Among all recorded road crossings, the proportion of females in the different anogenital swelling stages were as follows: 59.2% had no swelling (anestrous; scored “0”), 18.7% were partially swollen (scored “1”) and 22.1% were maximally swollen (i.e. in estrus; scored “2”). The observed distribution of these swelling stages when crossing in zone B was 38.5% for stage “0”, 28.2% for stage “1” and 33.3% for stage “2”. This distribution differed significantly from the average distribution (*χ* ^2^ test for given probabilities: *χ* ^2^_2_ = 6.805, *p* = 0.033). Thus, females crossed more frequently in the riskier zone B when they had partial and full anogenital swellings. In contrast, the observed distribution of females in the different swelling stages when crossing in zone A did not differ from the average distribution (62.7% for stage “0”, 17.1% for stage “1” and 20.2% for stage “2”; *χ* ^2^ test for given probabilities: *χ* ^2^_2_ = 1.310, *p* = 0.519). On average, 23.2% of female crossings occurred without any mature male in the (sub)group, while at least one male was present in 76.7% of female crossings. The observed distribution of maximally swollen females (stage “2”) was 5.1% of crossings without any male and 94.9% of crossings with male(s) (*χ* ^2^ test for given probabilities: *χ* ^2^_1_ = 10.693, *p* = 0.001), indicating that females in estrus generally crossed the road in the company of at least one mature male.

We found no influence of anogenital swelling status of females on their position, crossing gait, cautiousness or waiting time (null model comparison: *χ* ^2^ = 0.13, df = 2, *p* = 0.94; *χ* ^2^ = 5.54, df = 2, *p* = 0.06; *χ* ^2^ = 0.37, df = 2, *p* = 0.83; *χ* ^2^ = 2.47, df = 2, *p* = 0.29; respectively). However, the crossing gait model was close to our significance threshold (*p* = 0.06), indicating that maximally swollen females tended to cross at a slower pace compared to when they were non‐ or partially‐swollen (estimate = −1.21, z value = −2.28, *p* = 0.02). We also tested the influence of carrying a dependent individual on these behaviors in mature females, but no result was significant.

### 3.5 Hypothesis 2—Chimpanzee Risk Assessment

There was no effect of traffic intensity during crossings on individuals' waiting time (null model comparison: *χ* ^2^ = 2.88, df = 2; *p* = 0.24), cautiousness (null model comparison: *χ* ^2^ = 2.92, df = 2; *p* = 0.23) or protective behaviors (null model comparison: *χ* ^2^ = 0.06, df = 2; *p* = 0.97), but there was an effect of traffic intensity on gait (null model comparison: *χ* ^2^ = 70.54, df = 2; *p* < 0.0001). Chimpanzees crossed the road significantly faster when traffic intensity was higher.

**Figure 6:**
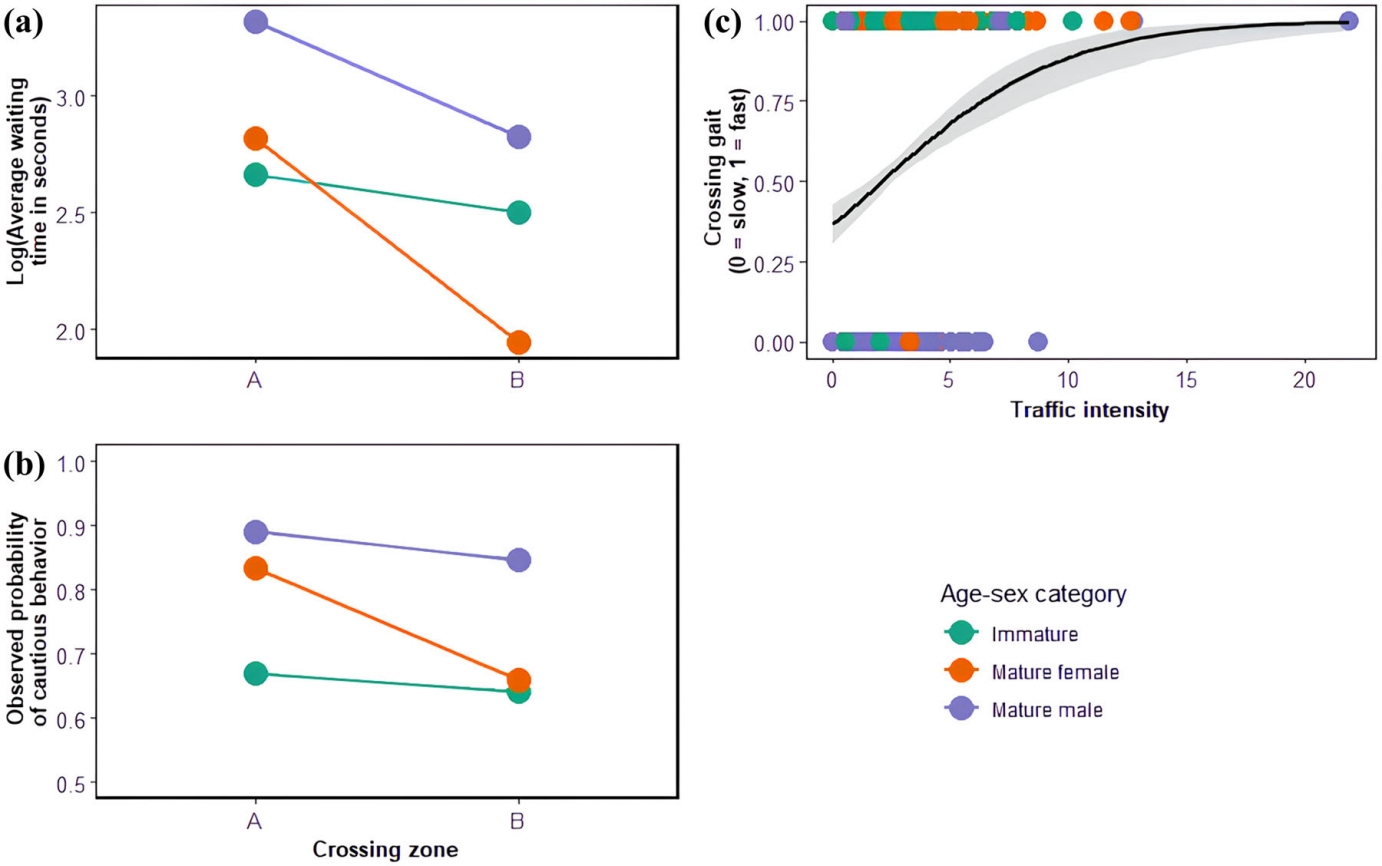
Relationships between the crossing zone and individual waiting times [log‐transformed, in seconds; (a)], and cautious behaviors (b) for each age‐sex category. The probability of crossing at a fast or slow gait is shown in relation to the traffic intensity during crossings (c).

There was an effect of crossing zone on waiting time (null model comparison: *χ* ^2^ = 7.85, df = 1; *p* = 0.0051) and cautiousness (null model comparison: *χ* ^2^ = 5.32, df = 1; *p* = 0.021). Chimpanzees waited for significantly less time when crossing in the riskier zone B and were less cautious (Figure 6). There was no effect of crossing zone on gait and protective behaviors (null model comparison: *χ* ^2^ = 0.89, df = 1; *p* = 0.34; *χ* ^2^ = 0.19, df = 1; *p* = 0.66; respectively).

We tested the influence of interventions by field assistants on cautiousness of the chimpanzees and found no influence (null model comparison: *χ* ^2^ = 1.71, df = 1; *p* = 0.19).

### 3.6 Hypothesis 3—Social Influences on Crossing Behavior

The mean crossing group size was 8.6 ± 4.7 (range 1–18) individuals, excluding infants. On average, chimpanzees crossed in 1.5 ± 0.8 (range 1–5) subgroups, composed of 5.1 ± 3.8 (range 1–18) individuals. Overall, 32.6% (42/129) of road crossing events comprised 2 or more subgroups, while in the remaining crossing events (87/129), the chimpanzees all crossed within 1 min of each other. Often the chimpanzees crossed in a loose manner with no linear progression discernible. Therefore, we could not always assign a precise progression rank to all the chimpanzees, except for the first and last individuals to step on or off the road. Notably, 34 of the 190 crossing (sub)groups included immatures crossing without their mother.

**Figure 7:**
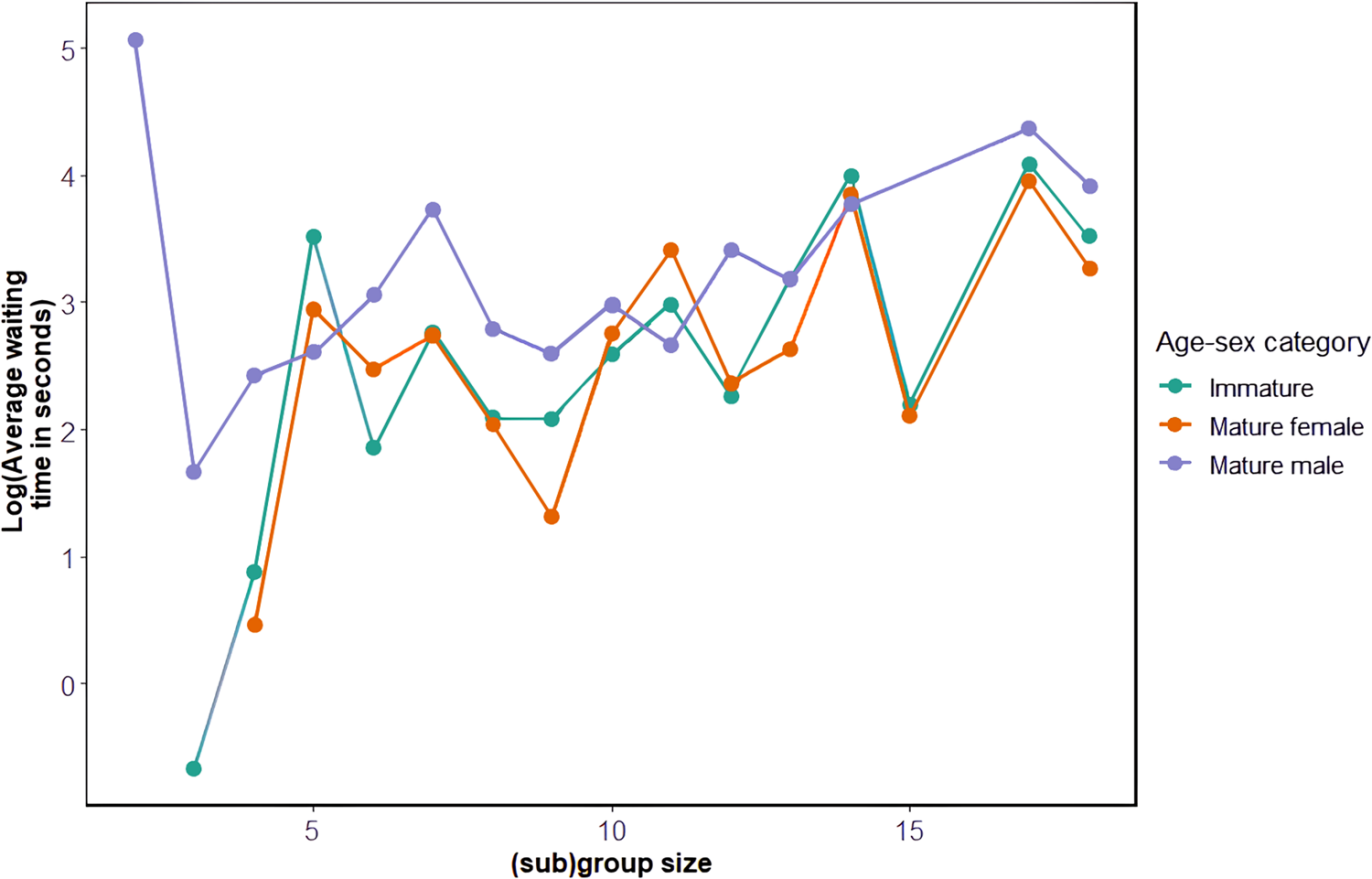
Line chart showing the effect of the crossing (sub)group size on individual waiting times (log‐transformed, in seconds) for each age‐sex category.

We found no effect of (sub)group size and proportion of mature males during crossings on cautiousness (null model comparison: *χ* ^2^ = 0.56, df = 1; *p* = 0.45; *χ* ^2^ = 0.67, df = 1; *p* = 0.41; respectively), and no effect of the proportion of mature males on crossing gaits (null model comparison: *χ* ^2^ = 1.25, df = 1; *p* = 0.26). However, we found a significant but slight effect of (sub)group size on crossing gait (null model comparison: *χ* ^2^ = 4.25, df = 1; *p* = 0.04): in larger groups the likelihood of individuals crossing at a fast pace decreased. There was no effect of the proportion of mature males on waiting times (null model comparison: *χ* ^2^ = 0.41, df = 1; *p* = 0.52), but there was a significant effect of (sub)group size on waiting times (null model comparison: *χ* ^2^ = 16.32, df = 1; *p* < 0.0001): individual waiting times increased in larger groups (Figure 7).

## 4 Discussion

In this study, which uses a larger dataset of behavioral observations than previous studies of road crossing in chimpanzees, we highlighted patterns of behavior that vary according to age and sex, level of risk, and group composition.

### 4.1 Does Road‐Crossing Behavior Vary According to Sex and Age Class?

Consistent with prediction (1.1), mature males were more likely to adopt forward and rearward positions than females and immatures. Younger chimpanzees predominantly occupied safer positions in the middle of crossing progressions. In support of prediction (1.2), males in general performed protective behaviors more frequently than others, such as waiting for conspecifics behind them, and adopting guarding postures while others crossed (Figure 5; Table S3–Video S4). Like previous studies at Bossou and Sebitoli (Hockings, Anderson, and Matsuzawa 2006; Cibot et al. 2015), these results confirm that chimpanzees can behave cooperatively to protect group members during “risky” situations. However, as Cibot et al. (2015) have already described, mothers did not always wait for their immature offspring or agree to carry them during crossings (Table S3, Video S5, Video S6; but see also Video S7 for the opposite behavior, i.e., maternal awareness of the vulnerability of dependent offspring). In 34 instances, immatures crossed in a (sub)group without their mother, including juveniles aged 4 and 5 years.

In support of prediction (1.3), mature males were recorded crossing the road more often than females and immatures. Like other small chimpanzee communities (Lehmann and Boesch 2004), the Bulindi chimpanzees are socially cohesive with parties containing 46% of individuals (excluding infants) on average (Satsias et al. 2022). However, parties in anthropogenic areas outside the forest, including croplands and the road, contain proportionally fewer females compared to parties inside the forest (Satsias et al. 2022). We therefore assume our sample of videos is representative of the composition of crossing groups and that field assistants were not more likely to record crossings involving mature males while missing crossings of females and immatures. Moreover, unlike females and immatures, males sometimes cross the road back and forth in short succession. This sex difference reflects the bolder behavior of male chimpanzees generally (Bertolani and Boesch 2008; Gilby et al. 2017; Haux et al. 2023) and specifically in human‐dominated environments like Bulindi (Hockings 2007; McLennan and Hill 2010; Satsias et al. 2022). These results also highlight how roads might reduce chimpanzee mobility (Garriga et al. 2019), particularly of females and immatures which may be reluctant to cross busy roads. Besides, given that chimpanzee societies are male‐philopatric (Goodall 1986; Nakamura et al. 2015; Boesch et al. 2019), greater road avoidance by females could lead to decreased female dispersal (Cibot et al. 2015) and increased inbreeding within chimpanzee populations (McCarthy et al. 2020). Long‐term studies at multiple sites crossed by wide and busy asphalt roads will be needed to assess the impact of these human infrastructures on female dispersal.

Contrary to prediction (1.3), mature males at Bulindi waited longer before crossing and were more cautious than females and immatures. Nevertheless, males did not generally exhibit signs of anxiety when surveying for traffic or pedestrians (such as bipedalism [Pierce 2009; Krief et al. 2014] or self‐scratching [Aureli and de Waal 1997; Hockings et al. 2007]). We suggest this difference reflects the greater willingness of males to be visible to road users, and thus more likely to be recorded checking traffic. In some primates, such as samango monkeys (*Cercopithecus albogularis*), age‐sex category has no effect on the number of glances (i.e. scans of the environment) made while crossing a canopy overpass (Linden et al. 2020), whereas in others, such as olive baboons (*Papio anubis*), adult males were described as checking traffic before crossing a road (Rowell 1969), similar to male chimpanzees at Bulindi. We further note that the traffic checking behavior we observed might sometimes simply reflect curiosity of road users rather than ‘cautiousness’.

**Figure 8:**
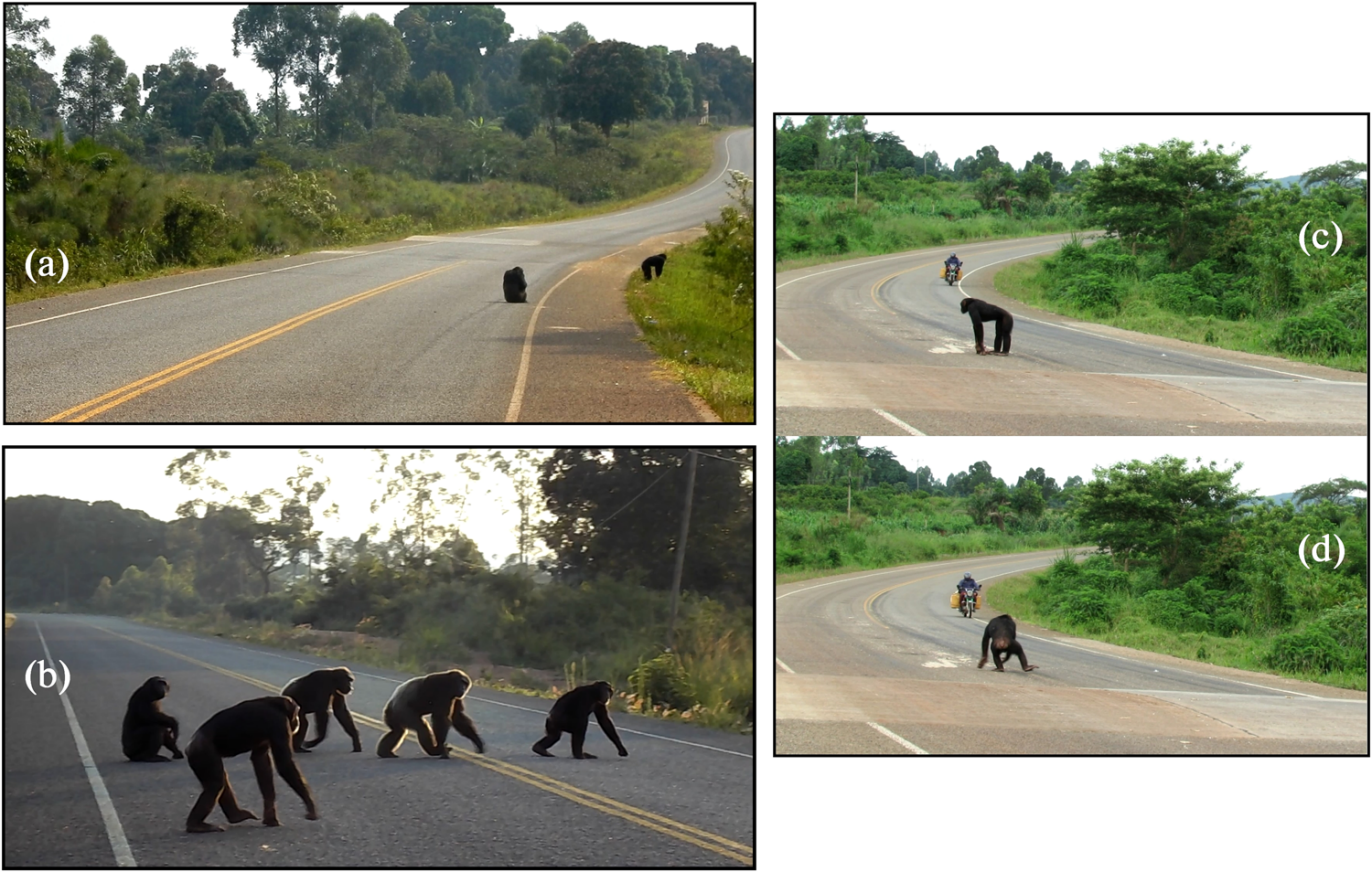
Screenshots showing mature male chimpanzees behaving “recklessly” during road crossings. MR (a) and MO (b) sitting in the middle of the road. (c) JK, standing alone in the middle of the road despite the rapid arrival of a motorbike, and (d) retreating at the last moment.

Unlike immature chimpanzees, which crossed faster than others as expected (prediction 1.3), mature males tended to cross at a leisurely pace, sometimes standing or sitting on the road without presenting signs of apprehension. At times they behaved “recklessly” by remaining visible at the roadside despite passing traffic (although we did not analyze such behavior specifically) or by moving away from vehicles at the last moment (Figure 8). Such behavior was not described in the previous studies of road crossing in chimpanzees. The difference in behavior with their closest relatives, the bonobos (*Pan paniscus*) described by Druelle et al. (2020), is particularly striking: when crossing a less risky dirt road, all bonobo individuals, including males, ran quickly or even leaped from tree to tree in response to a single passing cyclist. In the present study, mature female chimpanzees were intermediate in terms of crossing gait and cautiousness, and they were not more vigilant when carrying a dependent, contrary to our predictions and to what has been described previously in risky situations like crop feeding (Hockings 2007; Wallace and Hill 2012). These results suggest that Bulindi chimpanzees might not consider road‐crossing a high‐risk situation in many instances, except when traffic intensity is especially high. Although immatures generally crossed faster than mature individuals and occupied safer positions, they were less cautious. Immature chimpanzees could therefore be at highest probability of colliding with a vehicle (Supporting Information: Table S3 **–**Video S3).

Finally, consistent with prediction (1.4), we observed that females in estrus (i.e. maximally swollen) tended to cross more slowly, similar to males. Likewise, females were more likely to cross in the more dangerous zone B when they were swollen, even partially, compared to non‐swollen. These results align with previous observations (e.g. Matsumoto‐Oda 1999) that females tend to range with mature males during estrus. Indeed, Satsias et al. (2022) reported that maximally swollen females in Bulindi exhibited increased gregariousness, even in “riskier” anthropogenic parts of the habitat.

### 4.2 Do Crossing Modalities Vary According to Degree of Risk?

Contrary to prediction (2.1), we found no evidence that traffic intensity influenced when chimpanzees crossed the road. While traffic intensity during crossings showed moderate variation at different times of the day (Figure 3), the chimpanzees crossed most frequently at times that correspond to their main hours of activity, i.e., early in the morning and late in the afternoon, when they do most foraging (e.g. Goodall 1986; Carlson, Rothman, and Mitani 2013). Indeed, chimpanzees at Bulindi are rarely active in the early afternoon before ca. 4 pm (unpublished data). We acknowledge that our measure of traffic intensity has limitations because (1) it was assessed over short durations during crossings (video recordings with mean duration of 2.1 min; range 14 s‐16 min), (2) we likely missed some pedestrians or vehicles that were behind the camera and inaudible, and (3) recordings normally started once 1 or more chimpanzees had approached the roadside, whereas at times chimpanzees might have assessed traffic over longer periods before approaching the road. In zone A, they sometimes spent time in dense vegetation near the roadside before any individual crossed. In such instances, the chimpanzees might have been waiting for traffic intensity to reduce before deciding to cross; alternatively, they could have been resting and socializing out of view of passing road users. Unfortunately, we could not assess these possibilities since the chimpanzees were rarely visible at these times.

In agreement with prediction (2.2), chimpanzees—especially females and immatures—crossed most often in the safer zone A, which had improved visibility, lower vehicle speeds due to humps, and higher vegetation coverage on both sides compared to zone B. Hence, our results support the idea that chimpanzees choose where to cross to limit the risk of collisions or unexpected encounters with pedestrians. Nevertheless, this difference could also be explained by the distribution of habitat cover in their home range. The chimpanzees predominantly foraged around zone B because of the presence of cultivated fruit trees such as jackfruit, mango, and guava by homesteads (personal observations). Therefore, it is unlikely that food motivation explains why chimpanzees crossed more frequently in zone A. However, chimpanzees could access zone A with relatively little exposure to humans due to the vegetation cover south of the road (riverine forest, papyrus swamp and fallow fields; Figures 1c and 2). In contrast, to reach food sources north of the road in zone B, chimpanzees had to traverse open farmland near homes (covering a minimum distance of 500 meters from riverine forest to the southeast) and cross the road where there is little cover, or cross the road at zone A, where they were less conspicuous and could take advantage of vegetation cover on the northern side of the road as they travel to zone B. While a study of their daily travel and foraging routes would shed further light on this issue, the chimpanzees appeared to adopt the latter strategy most often, especially females. As noted above, Bulindi's females generally venture less often into “anthropogenic” parts of the habitat (including open farmland and village areas such as the surroundings of zone B) compared to males (Satsias et al. 2022). Thus, the sex difference in the relative frequency that males and females crossed the road in the safer versus more dangerous zone may relate more to females' perception of risk in exposed areas, rather than to their risk perception of the road per se.

Contrary to prediction (2.3), chimpanzees waited less time before crossing in the riskier zone B. This is probably attributable to the limited cover available in zone B, making chimpanzees reluctant to spend time in an exposed position near the road. Additionally, they exhibited less caution (e.g. less traffic checking) in zone B. While humans typically wait longer in areas with busier traffic and faster vehicles, they are also more likely to attempt riskier crossings (Hamed 2001; Simpson, Johnston, and Richardson 2003). Surprisingly, however, although vehicles could travel faster on this section of the road, the chimpanzees did not cross at a faster gait. Overall, Bulindi chimpanzees seem to perceive risk differently from those at Bossou, who waited longer before crossing a wider and presumably more dangerous road, compared to a smaller one, and generally crossed it more quickly (Hockings 2011).

**Figure 9:**
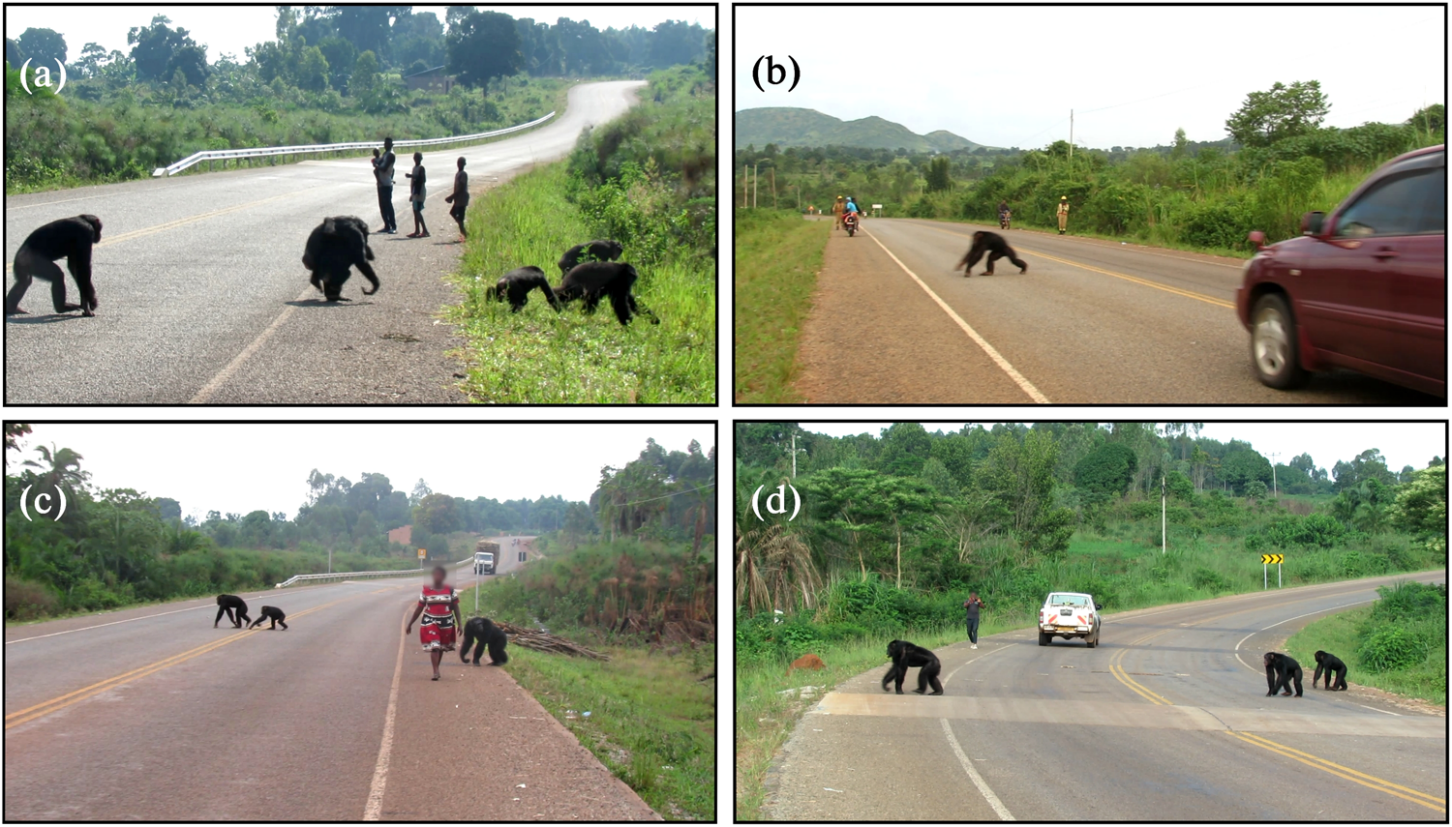
Video screenshots showing instances of close proximity between chimpanzees and road users during road crossings. (a) Mature males (JK, AR, MO) and MN's family crossing near onlookers. (b) JK narrowly avoiding a car, with nearby onlookers (electricity workers) and motorcyclists. (c) From left to right: JK, RO (immature male), and MO crossing very close to a pedestrian, with an oncoming truck. (d) Mature males (MO, AR, GD) crossing at a speed hump (boundary between zones A and B) in proximity to a car that has stopped in the road; the driver is approaching the chimpanzees to film them.

Nevertheless, the chimpanzees did cross faster when traffic intensity (especially motor vehicle traffic) increased, presumably to reduce the risk of collision. However, we found no variation in cautiousness or waiting time as a function of motor and non‐motor traffic intensity during crossings. At times chimpanzees seemed to wait because of nearby pedestrians or approaching vehicles (and sometimes showed avoidance behavior), while at other times they crossed in close proximity to people and vehicles (Figure 9; Table S3 and Videos S1–S3). Overall, the chimpanzees in Bulindi seem less prudent in their road crossing behavior compared to those in Bossou and Sebitoli, despite heavy traffic. At Bulindi, the chimpanzees have a long experience of crossing this road (McLennan and Asiimwe 2016) and may have developed tolerance (i.e. reduced reactivity; Čapkun‐Huot et al. 2024) to the risk it presents even though the danger has increased following the recent upgrades (tarmacking and widening; faster vehicles).

### 4.3 Does Individual Vigilance Vary According to Group Size and Composition?

As described in olive baboons in Ethiopia (Aldrich‐Blake et al. 1971) and in Sebitoli chimpanzees (Cibot et al. 2015), individuals in Bulindi frequently split into small subgroups within a larger crossing group. At Sebitoli, it was suggested that crossing in small subgroups was a strategy to enhance group protection on a road with high traffic intensity and short distances between vehicles. At Bulindi, the chimpanzees were more cohesive during crossings than the considerably larger Sebitoli community of around 80 individuals (i.e. subgroups were fewer and larger on average in Bulindi: 1.5 subgroups per crossing composed of 5.1 individuals versus 2.7 subgroups per crossing comprising 2.1 individuals in Sebitoli). While we are unable to compare traffic intensity at Bulindi and Sebitoli, this difference could reflect the fact that the small community at Bulindi is socially cohesive generally (Satsias et al. 2022), like small chimpanzee communities elsewhere (Lehmann and Boesch 2004).

In agreement with prediction (3.1) and similar to the Bossou chimpanzees (Hockings 2011), individuals at Bulindi waited longer in large crossing groups than in small ones, probably because they waited for others to cross before doing so themselves. The latency between the crossing of the first chimpanzee and a subsequent individual may reflect the latter's confidence that the leader chose a safe moment to cross; however, we were unable to investigate this possibility in the present study. In line with prediction (3.2), chimpanzees tended to cross more quickly in small groups than in large ones, contrary to those at Sebitoli (Cibot et al. 2015). Against expectation (3.3), however, the chimpanzees were no more cautious in small versus large groups, suggesting that they did not feel more vulnerable on the road in small numbers. Considering the Bulindi chimpanzees are habituated to researchers (McLennan et al. 2019), we hypothesize that the nearby presence of familiar field assistants meant the chimpanzees felt safer during the observed crossings, and therefore showed less caution than they might otherwise do when field assistants are absent. Once again, this overall lack of fear could also be explained by their long experience of crossing this road, even if it was previously untarmacked with less vehicle traffic (McLennan and Asiimwe 2016).

### 4.4 Conservation and Management Implications

We suggest there are probably regional differences in chimpanzees' responses to roads. Garriga et al. (2019) reported general avoidance of roads by chimpanzees in West Africa (Sierra Leone), where human population density is much lower than in parts of East Africa, i.e. around 50 persons/km^2^, versus > 150 persons/km^2^ in this study. In contrast, chimpanzees in Bulindi and elsewhere regionally (McLennan 2008; McLennan et al. 2021) have frequent contact with people, vehicles and roads, and mature males especially do not seem to avoid roads that cross their territories. Consequently, our findings, which suggest that Bulindi chimpanzees are responding to roads differently to what is described at other sites where anthropogenic impact is lower, testifies to the behavioral flexibility of chimpanzees (Hockings et al. 2015; McLennan, Spagnoletti, and Hockings 2017). Studies of chimpanzee road crossings in different habitats, and using larger datasets, are welcome to provide additional descriptions of the interactions between road users and chimpanzees and the factors influencing them, and to aid our understanding of risk perception and progression orders in chimpanzees.

Despite the social and behavioral adjustments shown by the Bulindi chimpanzees, the danger of these road crossings remains very high given the volume of traffic and speed of vehicles on the upgraded road (up to 120 km/h on some sections; personal observations). Additionally, the road could potentially limit female dispersal, as noted above. In western Uganda, chimpanzee habitats are subject to ongoing road and infrastructural development, driven in part by the demands of the oil industry (Mawejje 2018; Ogwang and Vanclay 2019; McLennan et al. 2021). It is therefore crucial to implement measures to mitigate the impact of current and future road construction and renovation on Uganda's chimpanzees and other wildlife.

We recommend that the road authorities install speed bumps along the stretch of road crossed by chimpanzees in Bulindi, and elsewhere where roads are being developed in chimpanzee habitats. Existing humps should be improved to enhance their effectiveness, for example by elevating them (see Antić et al. 2013) or by implementing double bumps (see Kiran, Kumar, and Abhinay 2020). Strict and regular police speed controls on roads passing through chimpanzee habitats could also help mitigate impacts on these endangered great apes (McLennan and Asiimwe 2016; Krief et al. 2020). These measures would also benefit the many pedestrians and cyclists who use these roads. In addition, studying the behavioral reactions of road users to chimpanzee crossings would be valuable for assessing the effectiveness of mitigation measures. In areas where humans and chimpanzees coexist, such as in western Uganda, we also recommend conducting awareness campaigns about chimpanzee road‐crossing behavior, including the age‐sex differences highlighted in our study, to help road users respond safely during encounters. This would allow local residents to better understand chimpanzee behavior on roads, and adjust their own behavior to limit the risk of collision or overly close encounters with the animals (Tellier et al. in prep). Finally, broadcasting a radio awareness campaign to encourage people to drive slower in areas frequented by chimpanzees, and to give them space to cross when encountered, would reach a wider population, including non‐local workers commuting along these roads and drivers of tourist vehicles—both of which are less familiar with chimpanzee road crossing behavior than most local persons.

## 5 Conclusion

This study offers valuable insights into the road‐crossing behavior of wild chimpanzees, highlighting variations based on age, sex, and group dynamics. While chimpanzees in Bulindi seem to perceive road crossings as dangerous and exhibit behavioral strategies to reduce risks of collision or close encounters with humans—as described in previous studies (Hockings, Anderson, and Matsuzawa 2006; Hockings 2011; Cibot et al. 2015)—overall they were less vigilant than we expected: their use of the road was related to their main hours of activity rather than low traffic intensity; they did not wait for longer before crossing or show more caution when traffic intensity was highest; juveniles sometimes crossed without their mothers; they often crossed in a “nonchalant” manner; and they were not more vigilant when crossing in small groups. These findings indicate that chimpanzees at Bulindi have developed behavioral tolerance toward the road (presumably through habituation‐like processes; Blumstein 2016), even though it was widened and paved shortly before the study began. Prior to its upgrade, the chimpanzees crossed this road for generations (McLennan and Asiimwe 2016). At the time of this study, they crossed the road several times a week on average (unpublished data). Their growing experience of crossing the upgraded road may mean they pay increasingly less attention to the risk it presents, potentially increasing the likelihood of a collision. In that respect, their behavioral tolerance of the road could ultimately prove maladaptive if it reduces individuals' fitness (see Čapkun‐Huot et al. 2024). The chimpanzees' overall lack of fear of the road is probably related to their long history of exposure to people and long familiarity with vehicles on the road (McLennan and Hill 2010; McLennan et al. 2019; Satsias et al. 2022), despite the fact the road has become busier and—from our perspective—more dangerous for them after tarmacking. Thus, it is crucial to raise awareness among local people and implement sustainable measures to mitigate collision risks, pollution, and zoonosis transmission on this road. Studying the behavior of other chimpanzee populations facing similar road crossings will help refine conservation strategies and inform best practices for road construction and renovation. Future research should also focus on longer‐term effects and broader geographic studies to better understand and mitigate the impacts of human infrastructure on chimpanzee populations (Videos S1, S2, S3, S4, S5, S6, S7).

**Video S1:** Chimpanzees crossing the road in close proximity to pedestrian onlookers.

**Video S2:** Chimpanzees crossing the road with busy traffic and close onlookers (electricity workers and motorcyclists), with JK narrowly avoiding a car (00:22).

**Video S3:** Chimpanzees crossing the road with busy traffic, with immature male MK narrowly avoiding a car thanks to the driver braking upon noticing the preceding chimpanzees (0:36).

**Video S4:** Chimpanzees crossing the road with pedestrians (visible and/or audible) and vehicle traffic, with MR displaying protective behavior towards GD who was limping from a temporary injury (00:56).

**Video S5:** Chimpanzees crossing the road with mature female MN refusing to carry her 4‐year‐old dependent offspring LC (00:05). Field assistant TS is standing at the roadside.

**Video S6:** Chimpanzees crossing the road with LL already carrying her 2‐year‐old infant GF and refusing to also carry her 5‐year‐old dependent offspring WD (00:37).

**Video S7:** LL crossed the road carrying her 2‐year‐old infant GF and showing protective behavior towards her 5‐year‐old dependent offspring WD (encouraging and waiting for her while crossing).

## Author Contributions

**Marie Tellier:** conceptualization (equal), data curation (equal), formal analysis (equal), methodology (equal), writing–original draft (equal), writing–review and editing (equal). **François Druelle:** data curation (equal), formal analysis (equal), writing–review and editing (equal). **Marie Cibot:** conceptualization (equal), methodology (equal), supervision (equal), writing–review and editing (equal). **Johnmary Baruzaliire:** investigation (equal), resources (equal). **Tom Sabiiti:** investigation (equal), resources (equal). **Matthew R. McLennan:** conceptualization (equal), investigation (equal), methodology (equal), project administration (equal), resources (equal), supervision (equal), writing–review and editing (equal).

## Conflicts of Interest

The authors declare no conflicts of interest.

## Supporting information

Supporting information.

## Data Availability

A full data set and the R code can be found at the Open Science Framework at https://osf.io/jvm6n/?view_only=d690dca35caf4e76a270708f3ced9193.
