# Supplementary material for: Running the Risk: Road‐Crossing Behavior in Wild Chimpanzees (Pan troglodytes) in an Anthropogenic Habitat in Uganda
Source: Am J Primatol. 2025 Feb 8;87(2):e70000. doi: 10.1002/ajp.70000 (PMC13020666; doi:10.1002/ajp.70000)
Supplement: Supplementary file 8 — Supporting information. [file AJP-87-e70000-s001.docx]

**SUPPORTING INFORMATION**

**Table S1.** Chimpanzee subjects with their year of birth, age class in the first and last year of the study, and maternal kin relationships. Males without living mothers are listed first by age, followed by adult females by age with their respective offspring. Names of females are italicized. Birth year is estimated for individuals born before 2012.

| **Name** |  | **Year of Birth** | **Age Class in 2018** | **Age Class in 2021** | **Note** |
| --- | --- | --- | --- | --- | --- |
| **Sylvester** | SL | 1984-1988 | Adult |  | Alpha male 2012-2019; disappeared December 2019 |
| **Murry** | MR | 1990-1992 | Adult | Adult |  |
| **Moses** | MO | 2005 | Adult | Adult | Alpha male from January 2020 |
| **Araali** | AR | 2009 | Sub-adult | Adult | Change of age class in 2021 |
| ***Maureen*** | MN | 1974-1980 | Adult | Adult |  |
| **Jack** | JK | 2009 | Sub-adult | Adult | Change of age class in 2021 |
| **Rohen** | RO | 2013 | Juvenile | Sub-adult | Change of age class in 2021 |
| ***Lucia*** | LC | 2016 | Infant | Juvenile | Change of age class in 2020 |
| ***Teddy*** | TD | 1978-1984 | Adult | Adult |  |
| **Ally** | AL | 2014 | Juvenile | Juvenile |  |
| ***Ellena*** | EL | 2019 | – | Infant |  |
| ***Mirinda*** | MD | 1990-1992 | Adult | Adult |  |
| ***Jemima*** | JM | 2005 | Adult | Adult |  |
| ***Jackie*** | JA | 2018 | Infant | Infant |  |
| **Gerald** | GD | 2009 | Sub-adult | Adult | Change of age class in 2021 |
| **Merrick** | MK | 2013 | Juvenile | Sub-adult | Change of age class in 2021 |
| ***Maria*** | MA | 2016 | Infant | Juvenile | Change of age class in 2020 |
| ***Leila*** | LL | 2000-2001 | Adult | Adult |  |
| ***Georgia*** | GG | 2013 | Juvenile | Sub-adult | Change of age class in 2021 |
| ***Wendy*** | WD | 2016 | Infant | Juvenile | Change of age class in 2020 |
| **Gift** | GF | 2019 | – | Infant |  |
| ***Kaije*** | KJ | 2009 | – | Adult | Migrating subadult first observed in Bulindi in December 2019,  Change of age class in 2021 |
| ***Kasangwe*** |  | 2021 | – | Infant |  |

Age-sex classes: infants (newborn to 3 years old); juveniles (⪰4 years old); sub-adults (⪰8 years old); adult males (⪰12 years old); adult females (⪰12 years old or at first birth, if earlier) (Sugiyama 2004). For behavioral analyses, late-subadults (10 years old by end of the study year) were lumped with adults and referred to as ‘mature individuals’ whereas young sub-adults (8-9 years old) were lumped with juveniles and referred to as ‘immature individuals’ – see **Methods**.

**Table S2.** Ethogram of behaviors used for video analysis in ©BORIS. Most definitions are taken from referenced articles or have been modified to suit this study (marked by an asterisk). Some behaviors, not clearly defined in the existing literature for chimpanzees, have been added and defined specifically for this study.

| **Behavioral category** | **Specific behavior** | **Definition** | **References** |
| --- | --- | --- | --- |
| **Mode of travel** | **Slow crossing gait** | Walking throughout the entire crossing. | Cibot et al. 2015 |
|  | **Fast crossing gait** | Running for at least part of the crossing, including intermediate-speed gait, i.e. “ambling”. | Cibot et al. 2015  Schmitt et al. 2006 |
|  | **Limping** | Limping for a part or throughout the entire crossing. |  |
|  | **Carried** | Dependent carried by its mother throughout the entire crossing. |  |
|  | **Part-carried** | Dependent carried by its mother for only part of the crossing. |  |
|  | **Not carried** | Dependent wanted to be carried but refused by the mother; therefore traveling on the ground. |  |
| **Progression order (Position)** | **Risky position** | Individuals flanking the crossing (sub)group: first and last chimpanzees to step on to the asphalt (including an individual crossing alone). | Cibot et al. 2015* |
|  | **Protected position** | Intermediate position between first and last. |  |
| **Protective behaviors** | **Reassurance gestures** | Gestures produced in reaction to recipient’s distress or solicited from another, such as touching, kissing or embracing. | Roberts & Roberts 2016 |
|  | **"Waiting for others" behavior** | Stopping or slowing while looking behind during or after crossing; or crossing back over when other chimpanzees haven’t followed (excluding dependents waiting for their mother). | Cibot et al. 2015* |
|  | **Guarding behavior** | Standing in a quadrupedal and alert posture on the road for more than 3s without moving while others cross. | Hockings 2011* |
| **Apprehensive and cautious behaviors** | **Traffic checking** | Rotation of the head between 45° and 90° to the right and/or left at least once. | Cibot et al. 2015 |
|  | **Traffic checking from tree** | Chimpanzee, having climbed a tree, watching the road before crossing. |  |
|  | **Retreat** | Turning back after starting to cross. |  |
|  | **Avoidance** | Chimpanzee at roadside moving away in response to approaching or passing vehicles or pedestrians. | Jaeger et al. 2005* |
| **Reckless behaviors** | **Stop** | Stopping on the road (standing or sitting), indifferent to other crossing individuals or road users. |  |
|  | **Displaying** | Charging fast or swinging rapidly on the road while pilo-erection, with or without vocalizations. | Scott 2013 |

**REFERENCES**

Roberts, A. I., and S. G. B. Roberts. 2016. “Wild Chimpanzees Modify Modality of Gestures According to the Strength of Social Bonds and Personal Network Size.” *Scientific Reports* 6: 33864. https://doi.org/10.1038/srep33864

Scott, N. M. 2013. “Gesture Use by Chimpanzees (*Pan troglodytes*): Differences Between Sexes in Inter‐ and Intra‐Sexual Interactions.” *American Journal of Primatology* 75: 555–56. https://doi.org/10.1002/ajp.22133

Sugiyama, Y. 2004. “Demographic Parameters and Life History of Chimpanzees at Bossou, Guinea.” *American Journal of Physical Anthropology* 124: 154–165. https://doi.org/10.1002/ajpa.10345

**Table S3.** Video excerpts showing Bulindi chimpanzees crossing the Hoima-Masindi road.

| **ID** | **Videos** | **Description** |
| --- | --- | --- |
| **V.S1** | 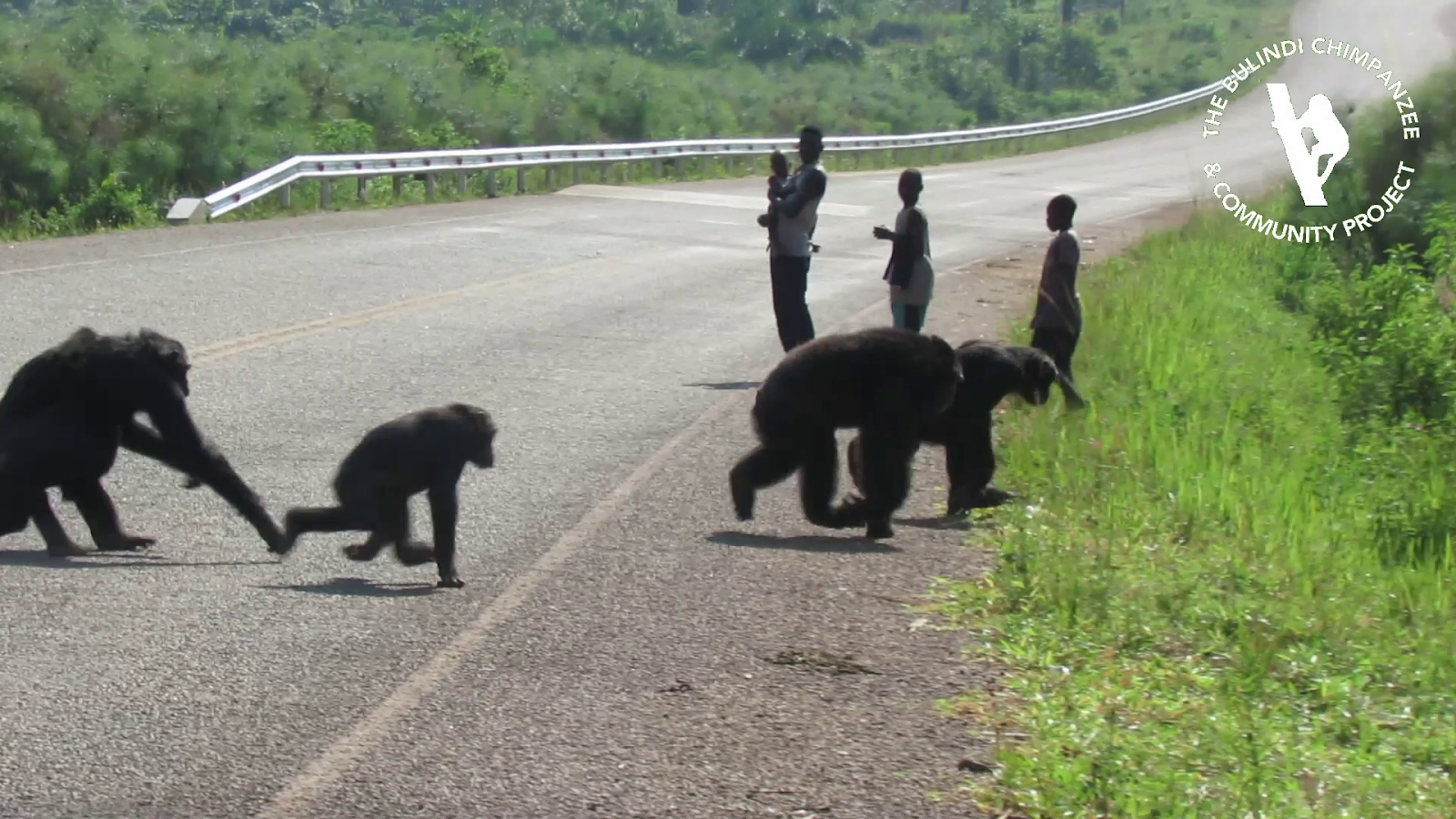 | Chimpanzees crossing the road in close proximity to pedestrian onlookers. |
| **V.S2** | 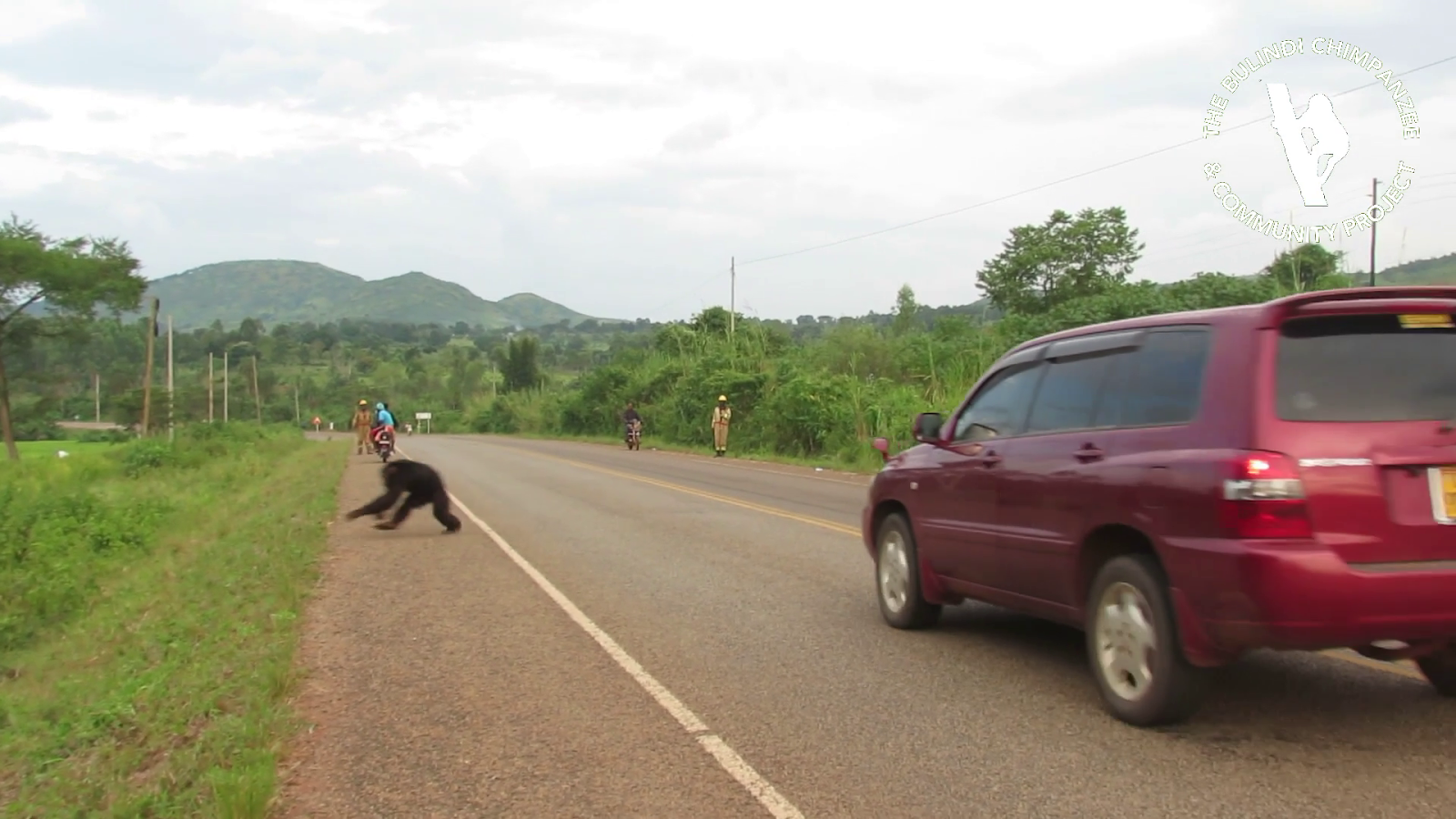 | Chimpanzees crossing the road with busy traffic and close onlookers (electricity workers and motorcyclists), with JK narrowly avoiding a car (00:22). |
| **V.S3** | 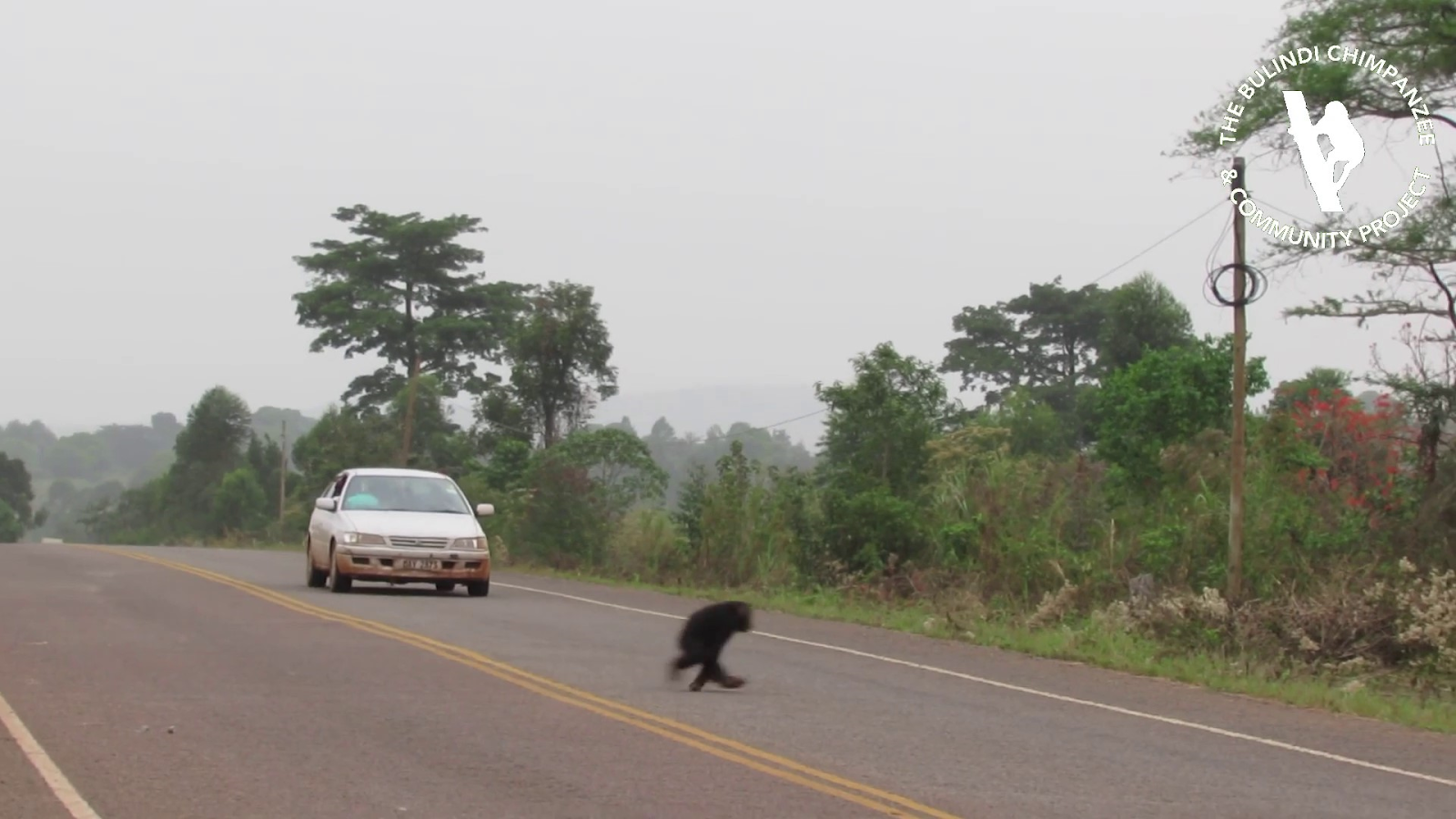 | Chimpanzees crossing the road with busy traffic, with immature male MK narrowly avoiding a car thanks to the driver braking upon noticing the preceding chimpanzees (0:36). |
| **V.S4** | 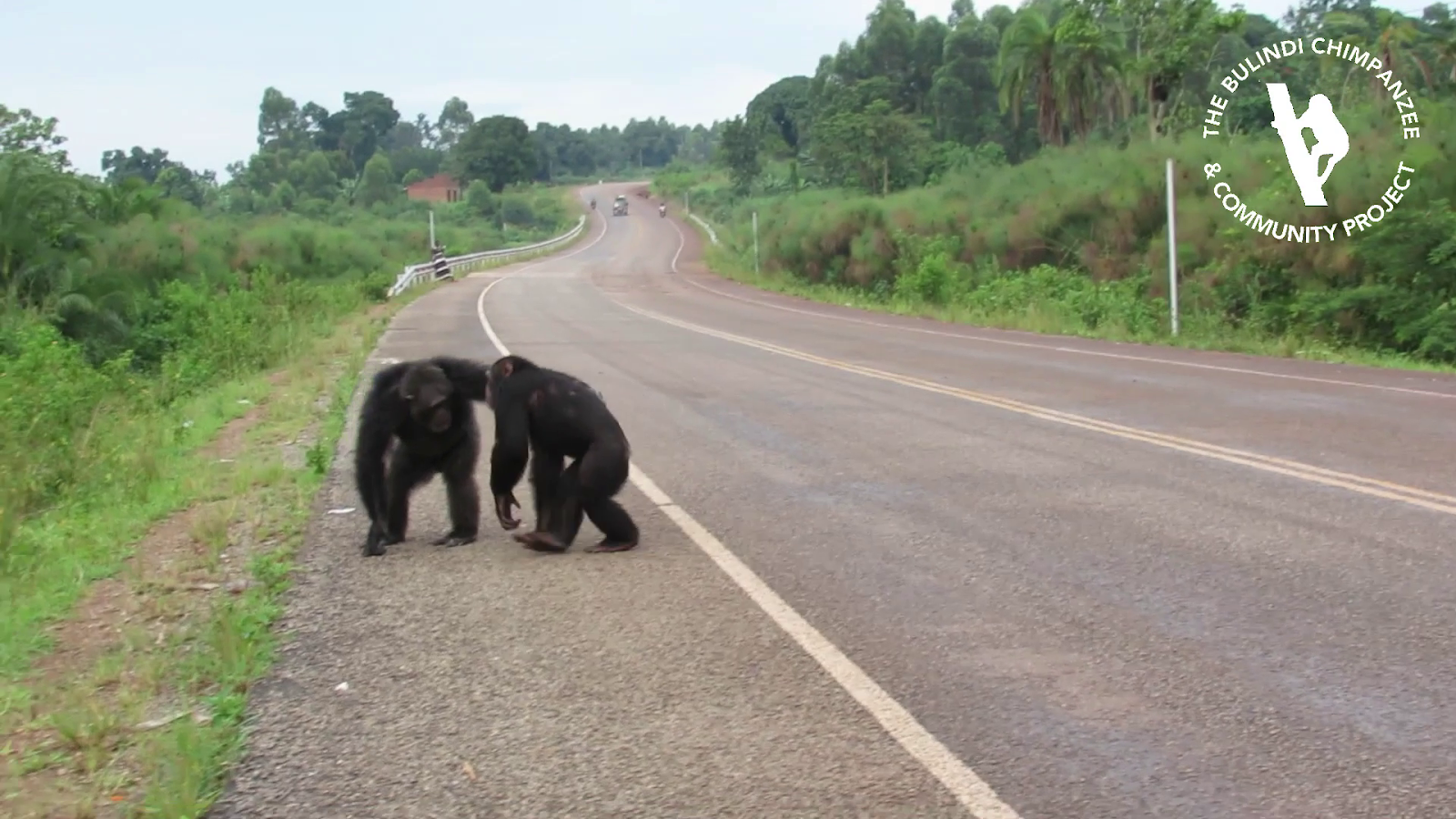 | Chimpanzees crossing the road with pedestrians (visible and/or audible) and vehicle traffic, with MR displaying protective behavior towards GD who was limping from a temporary injury (00:56). |
| **V.S5** | **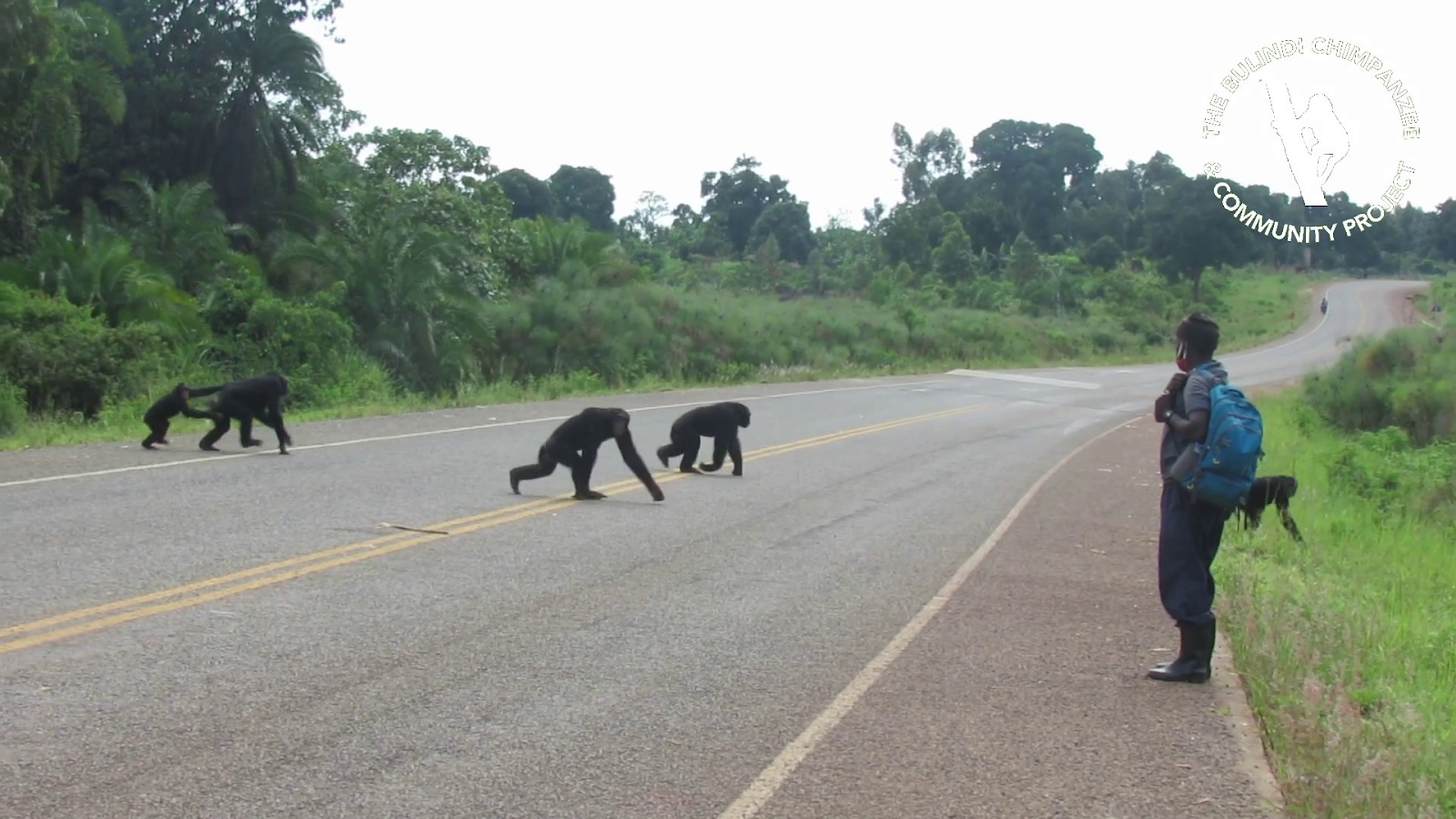** | Chimpanzees crossing the road with mature female MN refusing to carry her 4-year-old dependent offspring LC (00:05). Field assistant TS is standing at the roadside. |
| **V.S6** | **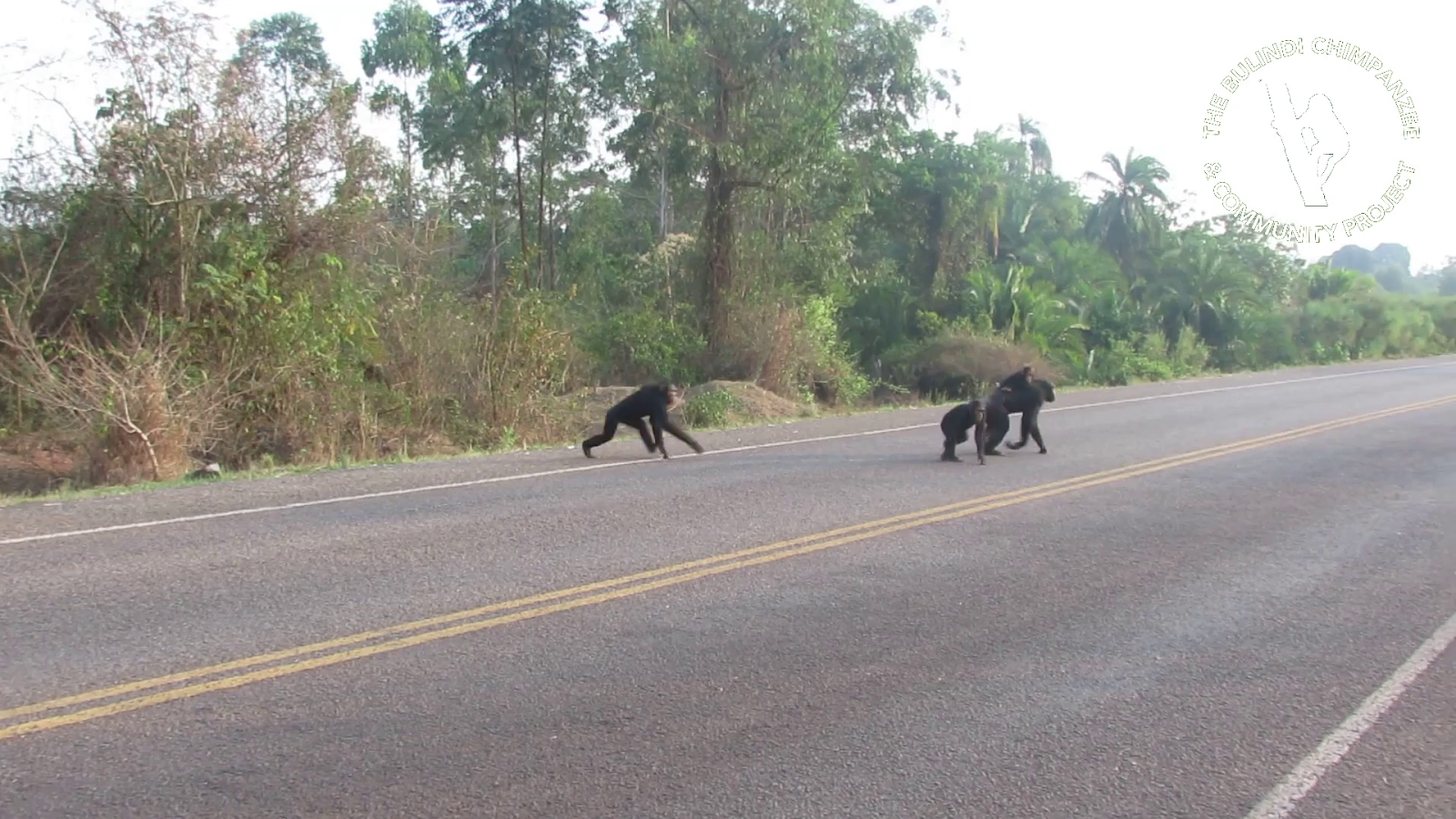** | Chimpanzees crossing the road with LL already carrying her 2-year-old infant GF and refusing to also carry her 5-year-old dependent offspring WD (00:37). |
| **V.S7** | 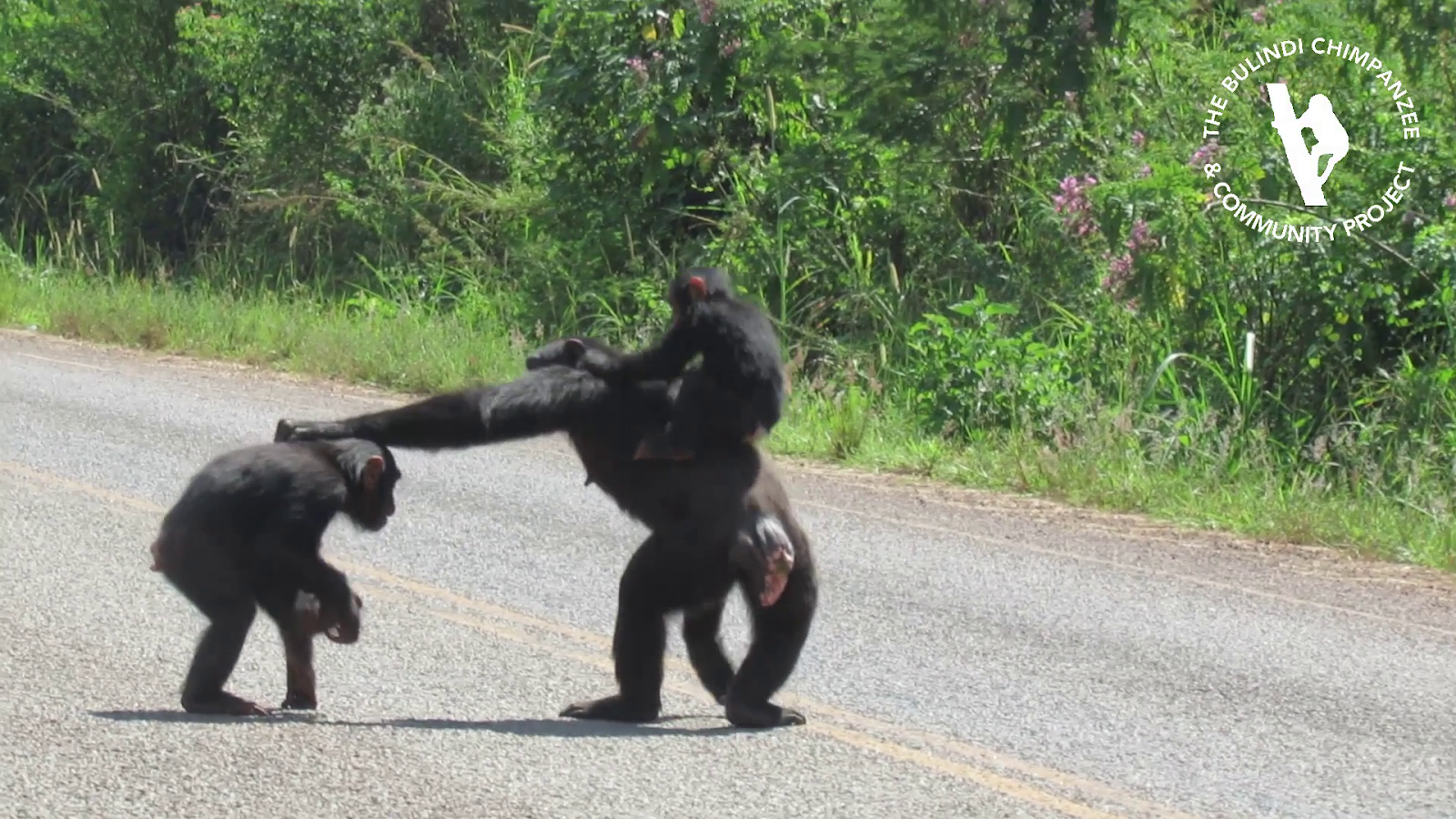 | LL crossed the road carrying her 2-year-old infant GF and showing protective behavior towards her 5-year-old dependent offspring WD (encouraging and waiting for her while crossing). |
